# Internet-enabled lab-on-a-chip technology for education

**DOI:** 10.1038/s41598-024-65346-0

**Published:** 2024-06-22

**Authors:** Tyler Sano, Mohammad Julker Neyen Sampad, Jesus Gonzalez-Ferrer, Sebastian Hernandez, Samira Vera-Choqqueccota, Paola A. Vargas, Roberto Urcuyo, Natalia Montellano Duran, Mircea Teodorescu, David Haussler, Holger Schmidt, Mohammed A. Mostajo-Radji

**Affiliations:** 1 Department of Electrical and Computer Engineering, University of California Santa Cruz, Santa Cruz, CA 95064 USA; 2 Department of Biomolecular Engineering, University of California Santa Cruz, Santa Cruz, CA 95060 USA; 3 Genomics Institute, University of California Santa Cruz, Santa Cruz, CA 95060 USA; 4 Live Cell Biotechnology Discovery Lab, University of California Santa Cruz, Santa Cruz, CA 95060 USA; 5 Centro de Electroquímica y Energía Química (CELEQ), Universidad de Costa Rica, San José, 11501 2060 Costa Rica; 6 Biotechnology, Universidad Católica Boliviana San Pablo, Santa Cruz de la Sierra, Bolivia

**Keywords:** Lab on a chip, Latinx education, Hispanic education, Internet of Things, Biology education, Electrical and electronic engineering, Bacteria

## Abstract

Despite many interventions, science education remains highly inequitable throughout the world. Internet-enabled experimental learning has the potential to reach underserved communities and increase the diversity of the scientific workforce. Here, we demonstrate the use of lab-on-a-chip (LoC) technologies to expose Latinx life science undergraduate students to introductory concepts of computer programming by taking advantage of open-loop cloud-integrated LoCs. We developed a context-aware curriculum to train students at over 8000 km from the experimental site. Through this curriculum, the students completed an assignment testing bacteria contamination in water using LoCs. We showed that this approach was sufficient to reduce the students’ fear of programming and increase their interest in continuing careers with a computer science component. Altogether, we conclude that LoC-based internet-enabled learning can become a powerful tool to train Latinx students and increase the diversity in STEM.

## Introduction

Several studies have shown the benefits of having a diverse workforce in the sciences, including increased citation number and impact of the publications, complementarity of skills, and better capacity to address health and social disparities^1,2^. Yet, science education remains highly unequal around the world and novel approaches are needed to provide quality education to students currently underserved in the sciences.

Previous work has shined lights into effective approaches for teaching complex topics to underrepresented students^3,4^. For Latinx students, for example, the most significant increase in conceptual understanding is observed when the projects are both collaborative and based on culturally and socially-relevant topics^3–6^, and when state of the art methods are used^4^. However, the implementation of this type of projects in the classroom is limited by high costs required to perform complex and novel experiments^3,7–9^. Novel strategies that connect laboratory equipment to the cloud through the Internet-of-Things (IoT)^7,10–13^ have the potential to scale the application of experimental education and reach underserved communities throughout the world^3^. Importantly, combining these approaches with local curricula has been shown to have similar learning outcomes and increase in STEM identity as in person instruction^3^.

Over the past decade, advances in lab-on-a-chip (LoC) technology have allowed the automatization of several laboratory techniques, especially molecular diagnosis and spectrometry-based characterization and quantification of molecules^11,14–16^. Due to their small size and nanoscale volumes required to perform experiments, LoC technologies can drastically reduce the costs of performing experiments^17,18^. LoC devices are very flexible, usually allowing the users to perform several experiments on the same chip architecture. For example, chips designed for polymerase chain reactions can, in principle, amplify genes of any disease or gene of interest^19^. Moreover, LoC technologies can be programmed and operated in standard or custom-made programming languages allowing for systematic movement of fluids and other functionalities. The combination of these properties makes LoC technologies powerful tools for experimental learning. However, despite all these advantages, LoC devices have rarely been implemented in the classroom, and when so they are in the context of teaching chemistry and microfluidics^17,18,20^. Their power as tools to train life sciences students in engineering concepts has not been explored.

Here we take advantage of IoT-enabled LoC devices to train Latinx life sciences students using context-aware assignments. This approach allowed us to reach students in Santa Cruz de la Sierra, Bolivia, while the experiments were performed over 8600 km apart, in Santa Cruz, California. Using water contamination testing as an inspiration, we used this methodology to expose students to introductory concepts in computer programming and show that the majority of them can rapidly acquire basic skills to write scripts to control LoC devices. We further demonstrate that this approach led to an increased comfort with programming, and a higher interest in undergoing computer science training. Altogether, we propose IoT-enabled LoC assignments as an efficient approach to democratize access to computational biology training and increase diversity in STEM.

## Results

### An internet-enabled LoC platform allows for remote control of fluidics experiments

**Figure 1:**
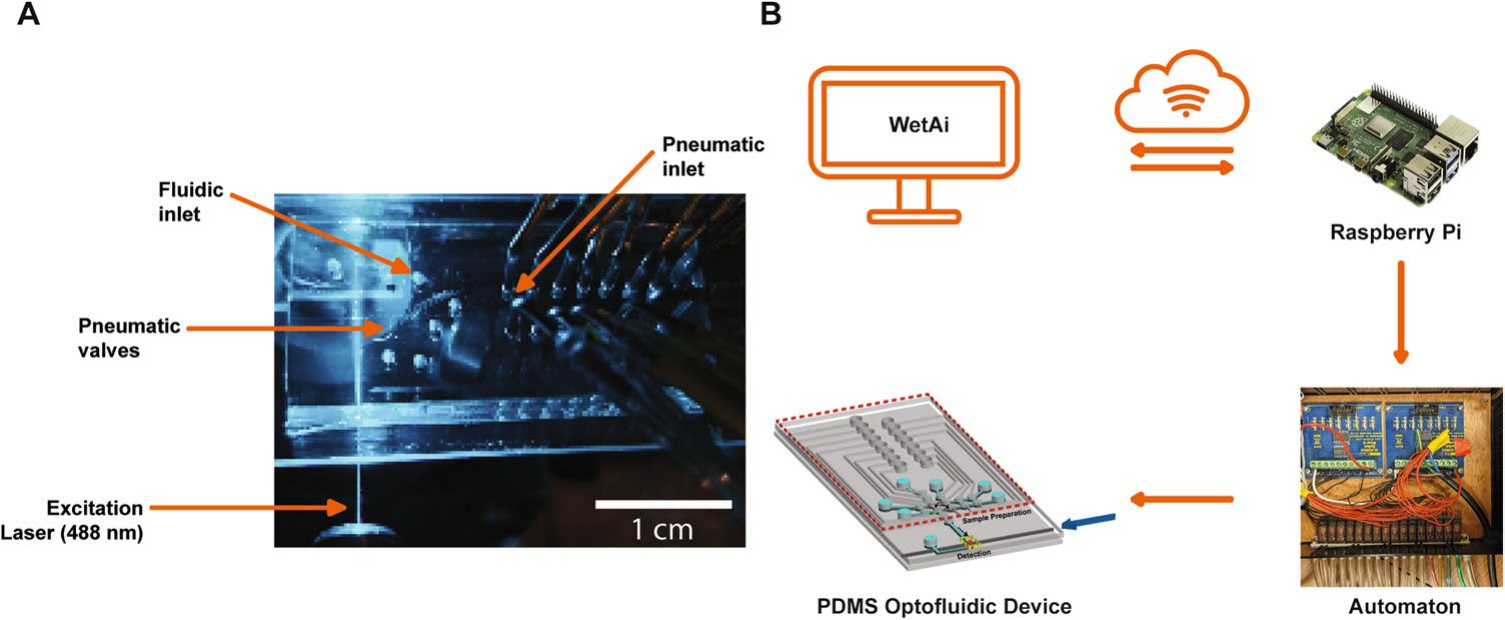
Internet enabled lab-on-a-chip platform. (**A**) The polydimethylsiloxane (PDMS) LoC optofluidics device combines sample preparation and optical detection capabilities. The chip contains pneumatically-operated “lifting-gate” valves that can be controlled to move and mix liquids. An external laser is coupled to the chip via a solid core PDMS waveguide, enabling optical excitation of fluorescent biomarkers. (**B**) A custom written script can be uploaded from a remote location to the cloud and received by a local IoT device. An interpreter program decodes the script and operates pneumatic valves in the PDMS optofluidic device for sample preparation and optical detection.

We developed an internet-enabled open-loop LoC platform, detailed in Fig. 1. In this platform, the LoC system, combining sample preparation and optical detection, was located within an optical setup at the University of California Santa Cruz. In this setup, an optical fiber carrying 488 nm excitation light was aligned to the solid-core excitation waveguide. Fifteen pneumatic lines connected an electronics box (Automaton) to the LoC, enabling remote pneumatic actuation and thus fluid manipulation and sample preparation.

As described in the methods section, any remote device accessing the local Raspberry PI device can conduct sequences of valve actuation to manipulate and move fluids through the device. We have included all the related code in https://github.com/braingeneers/IOT_Education_Lab_on_Chip_Paper. This platform is widely applicable for multiple experiments as the chip architecture itself can be altered as a result of the easy reconfigurability and rapid fabrication of PDMS devices. Additionally, the wavelength coupled to the excitation fiber can be changed, allowing for detection of different and multiple fluorescently labeled biomarkers. Several applications can be envisioned, ranging from traditional immunohistochemistry using antibodies, to microorganism detection. The ability to use LoC technologies in a variety of topics make them ideal tools for context-aware learning. Furthermore, the versatility of this tool allows it to reach a multitude of populations from high school students as a gateway into scientific disciplines to graduate students and researchers who need to use this tool for research. Moreover, the simplicity of the interpreter program allows this tool to be used as a first exposure to programming.

### Recruited Latinx life sciences students have little programming experience as part of their training

To test the impact of our IoT-enabled LoC device in the classroom, we recruited undergraduate biotechnology students at the Universidad Católica Boliviana San Pablo (UCB). This university is the only one to offer 5-year degrees in biotechnology in Bolivia (4 years instructional and 1 year internship and thesis writing). The biotechnology curriculum at UCB does not have required courses that expose students to introductory programming concepts. The students were part of two iterations of the course, which was taught in two different academic years. In one iteration, which we referred to as the control group, the class only had a lecture component. On the other hand, in the experimental group, the class had lectures and a practical programming assignment. The control group had 34 students, while the experimental group had 42.

**Figure 2:**
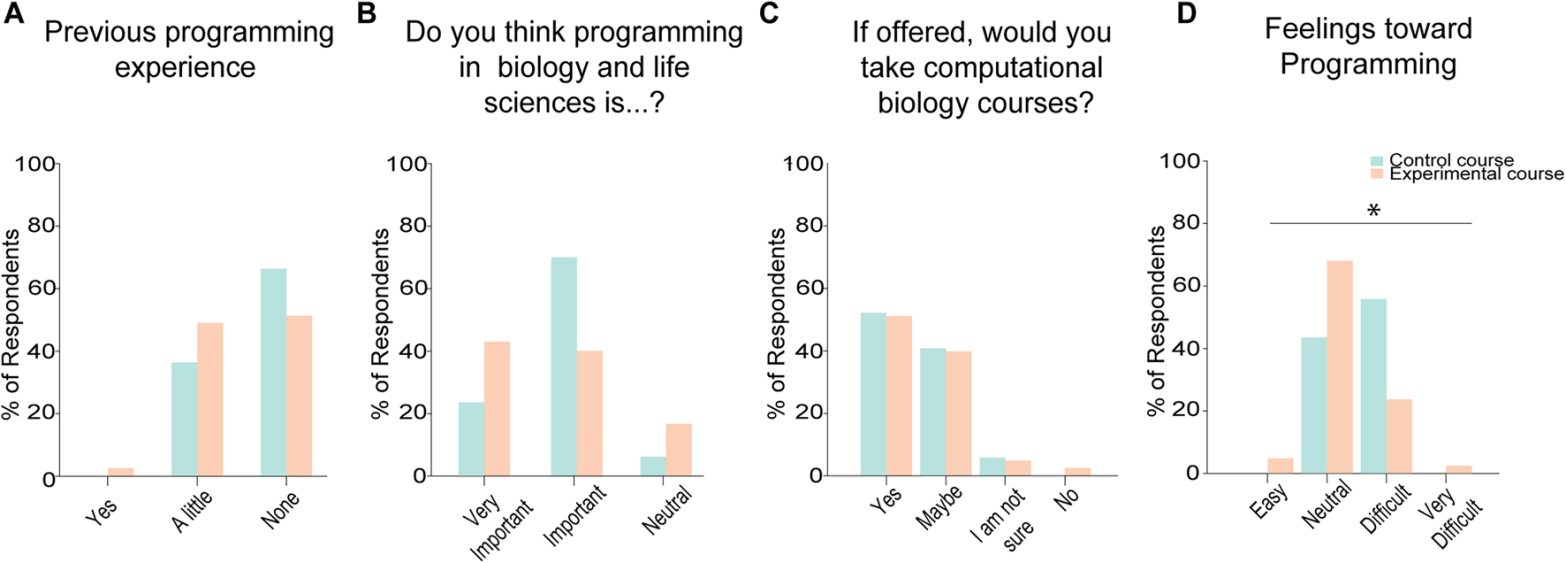
Recruited students had little programming experience. (**A**–**D**) Distribution of answers to pre-course survey questions. (**A**) Previous programming experience. (**B**) Perceived importance of programming in biology and life sciences. (**C**) Desire to take computational biology courses. (**D**) Feeling towards programming. n = 34 students in the control group and 42 students in the experimental group. * = *p* < 0.05.

Because computer programming is not part of the Bolivian high school curriculum, the large majority of biotechnology students have not had previous programming experience. Nevertheless, we asked the students their level of programming experience in any language. In the control group, 67.6% of the students reported that they had never programmed before, while 32.4% of the students had minimal programming experience. Similarly, in the experimental group, 69% of students responded that they have never programmed before with the remaining 31% reporting that their previous experience with programming was minimal (*p* > 0.05) (Fig. 2A).

Given their lack of programming experience, we asked the students whether they thought that programming was important for a career in biology and life sciences. Strikingly, we found that the large majority of students (94.1% in the control group and 83.4% in the experimental group) thought that programming was important, with 23.5% of students in the control group and 42.9% of students in the experimental group saying that it was “very important”, and 70.6% of students in the control group and 40.5% of students in the experimental group saying it was “important” (*p* > 0.05) (Fig. 2B). The majority of students (52.9% in the control group and 52.4% in the experimental group) said that they would take a computational biology course if it was offered by their university (*p* > 0.05) (Fig. 2C). We then asked the students whether they thought programming was easy or difficult. In the control group, 55.9% of the students felt that programming was difficult, while 40.1% felt neutral. In the experimental group, the large majority (69%) felt neutral on the difficulty of programming (*p* < 0.05 when compared to the control) (Fig. 2D), independently on whether they had previous programming experience or not (*p* > 0.05). Altogether we conclude that even though the majority of the students lack previous programming exposure, they understand the importance of programming in their careers.

Given their thoughts on programming, we asked the students how they perceived the representation of Latinx in the fields of bioengineering, as well as computational biology. We found that students thought that Latinx were underrepresented in both fields (Supplemental Fig. 1A,B). Specifically, 67.2% of students in the control group and 71.4% of students in the experimental group believed that Latinx scientists were underrepresented in computational biology (*p* > 0.05) (Supplemental Fig. 1A), while 64.7% of students in the control group and 66.7% of students in the experimental group thought that Latinx scientists were underrepresented in bioengineering (*p* > 0.05) Supplemental Fig. 1B). We then asked the students their thoughts on what are the obstacles to continue a career in computational biology. Overwhelmingly, the students said that lack of exposure is the main obstacle (44.1% control; 45.2% experimental) (Supplemental Fig. 1C). Other popular opinions included fear of programming (8.8% control; 19% experimental), and that lack of teacher training (17.6% control; 11.9% experimental) (Supplemental Fig. 1C).

Understanding that the students had little programming exposure, we asked them whether they would consider doing doctoral studies in computational biology after they finished their degree. In the control group, 32.4% of the students said “Maybe” while 52.9% of the students said they would consider doing a doctorate in computational biology, although it would not be their first choice (Supplemental Fig. 1D). On the other hand, in the experimental group, 45.2% said “Maybe”, while 38.1% of students said that they would consider doing a doctorate in computational biology, although it would not be their first choice (Supplemental Fig. 1D). Only 2 students in the control group (5.9%) and 2 students in the experimental group (4.8%) said that a doctorate in computational biology would be their first option, while 3 students in the control group (8.8%) and 5 students in the experimental group (11.9%) said they would “absolutely not” consider performing doctoral studies in this field (*p* > 0.05) (Supplemental Fig. 1D).

**Figure 3:**
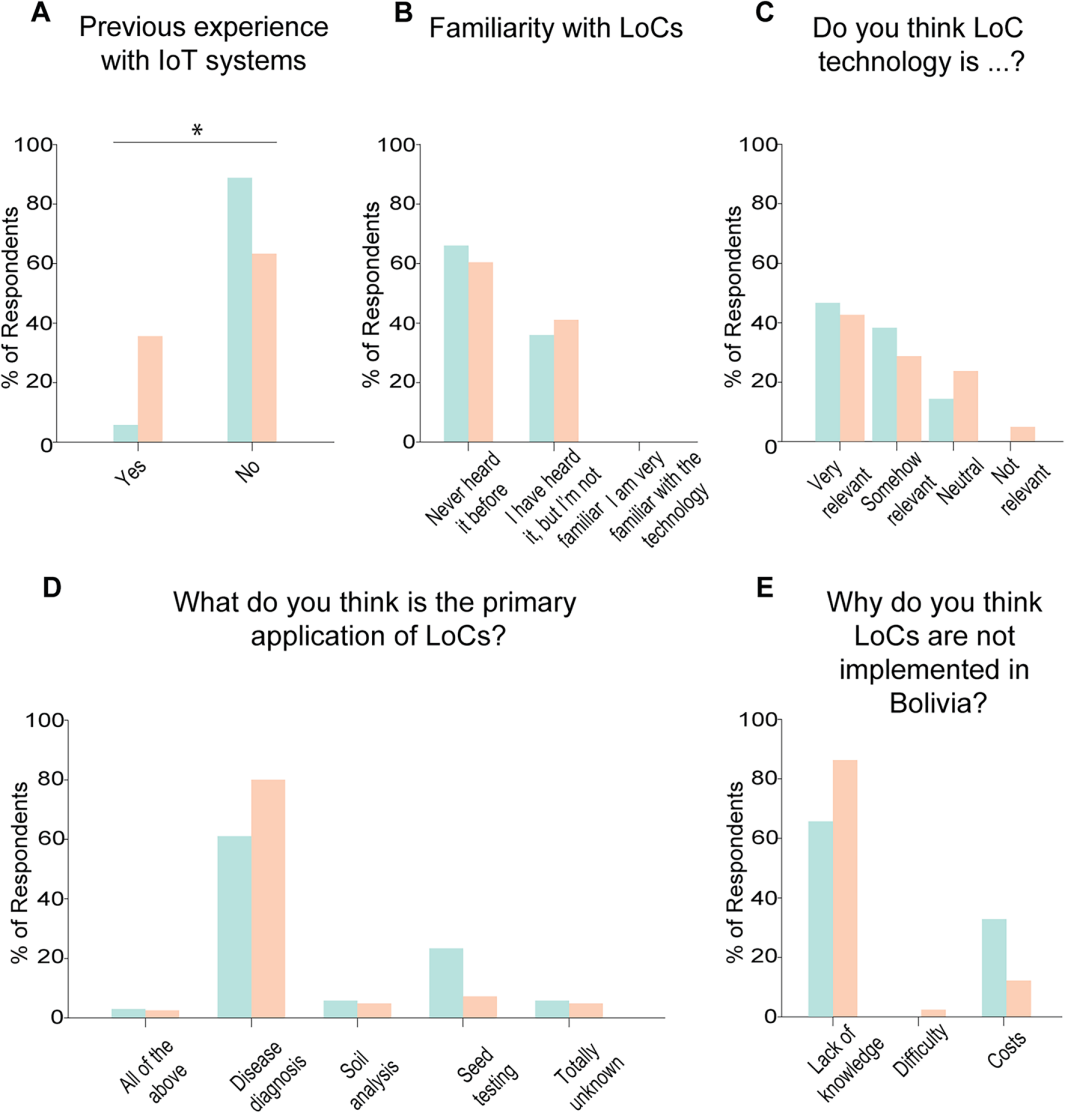
Recruited students had little exposure to LoC systems. (**A**–**E**) Distribution of answers to pre-course survey questions. (**A**) Previous experience using IoT systems. (**B**) Familiarity with LoC systems. (**C**) Perception of relevance of LoC systems. (**D**) Perception of applications of LoCs. (**E**) Perceptions of difficulties for application of LoC technologies in Bolivia. n = 34 students in the control group and 42 students in the experimental group. * = *p* < 0.05.

Previously, we used IoT-enabled microscopes to complement an introductory to biology course at the Universidad Católica Boliviana San Pablo^3^, and therefore a subset of these students have previously worked with our group, although none had worked with LoCs. To understand whether the students were familiar with IoT-enabled technologies we asked them if they had a previous experience using these IoT devices in the laboratory setting (Fig. 3A). We found that 94.1% of students in the control group and 64.3% of the students in the experimental group had no previous experience (*p* < 0.05) (Fig. 3A). When asked about their familiarity with LoC technologies, 64.7% of the students in the control group and 59.5% of the students in the experimental group said that they never heard of LoC technologies before, while the rest said that they have heard of LoCs before but they were not familiar with the technology (*p* > 0.05) (Fig. 3B). No student in any of the groups said that they were familiar with LoC technologies (Fig. 3B). We then asked the students if they thought that LoCs were easy or hard to use. The overwhelming majority (82.4% control; 81% experimental) of students thought that LoCs were hard to use (*p* > 0.05) (Fig. 3C). Interestingly, most (61.8% control; 81% experimental) of the students thought that the primary use of LoCs was disease diagnosis (Fig. 3D). When asked their thoughts on why LoCs are not implemented in Bolivia, 64.7% of students in the control group and 85.6% of the students in the experimental group said that it was because of lack of knowledge, while 32.4% of students in the control group 11.9% of the students in the experimental group thought it was due to high costs (Fig. 3E).

We then asked the students if they were familiar with some of the reagents that were going to be used in our project. Specifically, we asked students if they have ever used PDMS, the primary ingredient to make our LoCs. Most students (82.4% control; 76.2% experimental) said they never used PDMS before, while 14.7% of students in the control group and 21.4% of the students in the experimental group were unsure (*p* > 0.05) (Supplemental Fig. 2A). Most students (82.4% control; 64.3% experimental) said they had never worked with live bacteria before (*p* > 0.05) (Supplemental Fig. 2B). Finally, we found that the majority of students (61.8% control; 71.4% experimental) had previously visualized DNA (*p* > 0.05) (Supplemental Fig. 2C). Altogether, we conclude that while the students have had little exposure to programming and LoC technologies, they acknowledged the importance of the topics in their careers. Furthermore, we can conclude that the cohorts of students had knowledge and familiarity with the technologies used in this activity.

#### Development of a LoC-based context-aware curriculum

**Figure 4:**
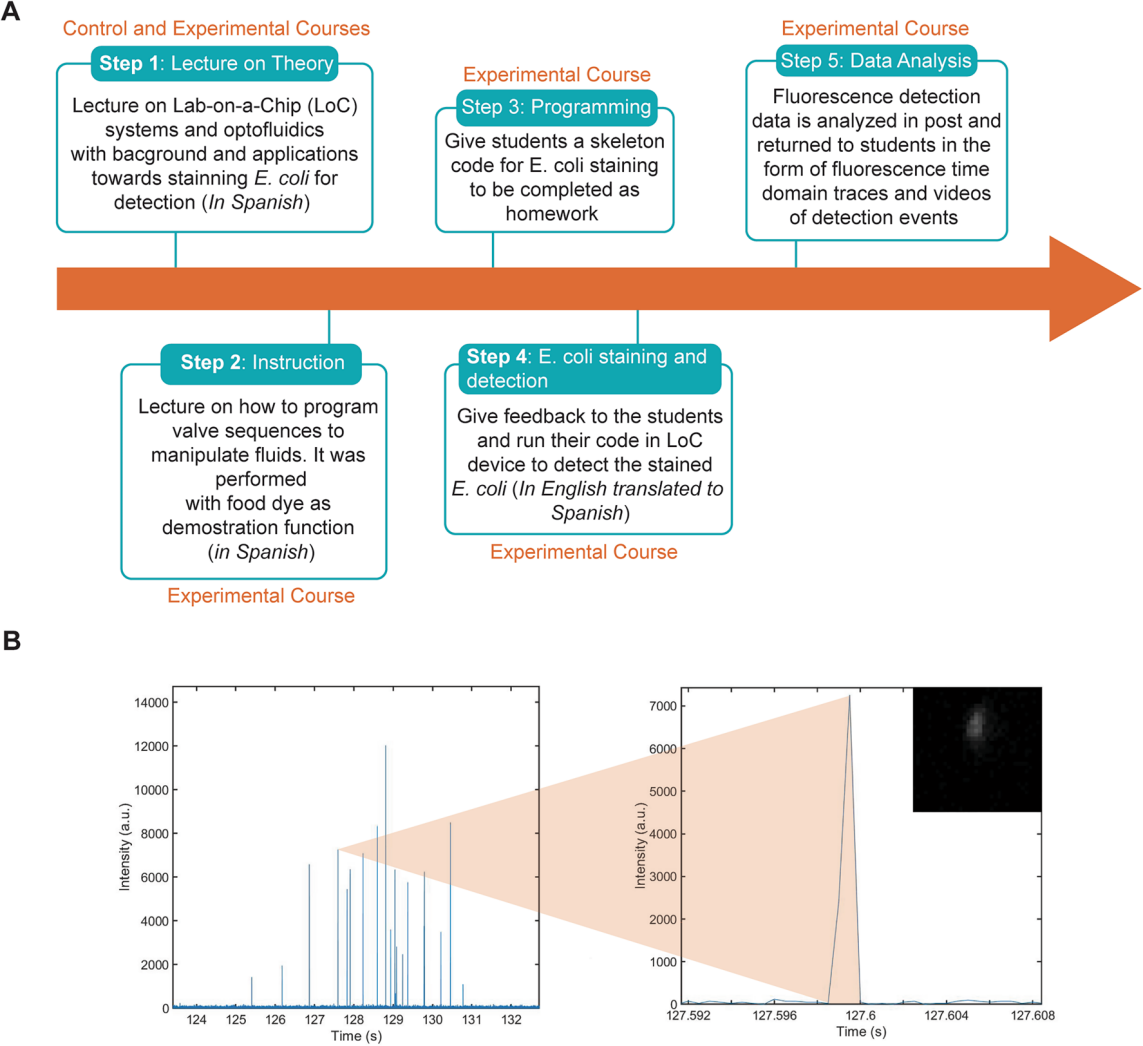
Context-aware remote training using LoC systems. (**A**) Training workflow. In the first lecture (taught to both groups) the students are taught the theory of LoC systems and their applications to pathogen detection. In the second lecture (taught only to the experimental group), the students are taught how to program LoC systems. Both lectures are taught in the students’ native language. The students in the experimental group are then assigned a project detecting bacteria in water samples. They then complete the assignment, upload their code and receive feedback. The LoC system is then run with their code and the data is produced, analyzed and returned to the students. (**B**) Example of detection of *E. coli* in the samples of one of the groups. Left: Detection of *E. coli* over 8 s. Right: Individual peak detected and corresponding *E. coli* bacterium that emitted the peak.

We designed a training module (Fig. 4) that was based on a common issue in Bolivia and the developing world: water quality. Bolivia is a country that has a long and complicated history of water accessibility^21^. Specifically, in the early 2000s, major protests in Cochabamba, Bolivia, known as the “water war”, forced the retreat of multinational water companies and the creation of public water industries^21^. Since then several governmental policies have focused on the establishment of water as a basic human right^21^.

As per water quality, previous work has tracked the water quality in urban and local populations in all 3 major regions of Bolivia: the lowlands, the valleys and the highlands^22^. This work revealed a significant increase in water contamination from the water source to the drinking cup, using *Escherichia coli* (*E. coli*) presence as a readout of fecal contamination^22^. Importantly, it was determined that 64% of the water samples tested had *E. coli* contamination^22^.

Given the history of both poor accessibility to water and high water contamination rates in the country, we used water testing as a gateway into teaching. We aimed to introduce the students in the experimental group to programming by asking them to detect individual *E. coli* cells in water samples. To accomplish this task, the students were expected to program LoC devices and perform staining of *E. coli* using the dye SYBR Gold, a highly-sensitive cyanine dye that intercalates with DNA^23^. As part of the process, the students needed to program the valves of a LoC device to first mix the water sample with the dye, then rotate the bacteria and the dye mixture through multiple valves to accomplish homogenization, then incubate the mixture and then carry it to the optical detection device. The task could only be accomplished if the correct sequence of valves can be precisely opened and closed, therefore requiring a high level understanding of the grid and the mechanisms of operations of the LoC device.

In preparation for the project, we lectured the students via Zoom. All lectures were given outside of their official classroom times (Fig. 4A). The lectures were conducted in Spanish, the native language of the students, by M.A.M.-R., who is a native Spanish speaker. We considered that teaching in Spanish as important, as it has been previously shown that science communication is more effective when done in the students’ native languages^24^. In the first lecture, which was given to both groups, we introduced the students to the concept of LoC technology and the specifics of the custom-made device that we made, including its fabrication methods and previous applications of the technology (Fig. 4A). In the second lecture, which was given only to the experimental group, the students were instructed on the various coding elements used to program sequential valve operation for manipulation of fluids through the chip (Supplemental Fig. 3). Lecture material included specific examples as well as a live demonstration of how the chip can be used to mix red and blue food dye as a proxy for illustrating the mixing of assay reagents (Supplemental Video 1).

At the conclusion of the second lecture, the students in the experimental group received a redacted version of a functioning code designed to mix *E. coli* and fluorescent staining dye together in the valves (Fig. 4A). Their homework was to complete the redacted portions of this code (Supplemental Fig. 4). Participating students in the experimental group were combined into groups of 3 to 4 students and given 4 days to complete their homework. Each group signed up for a one hour time slot to remotely run their *E. coli* staining protocol. During this time, students received feedback on their homework, ran their mixing sequence, and then had their final solution passed through the optical detection region for fluorescence detection (Fig. 4A,B). These meetings were led by T.S. and M.J.N.S. who are experts in LoC systems. The meeting was in English with simultaneous translation to Spanish by J.G.-F., S.H. or S.V.-C., all of whom are native Spanish speakers. In preparation for each session, 2 μL of *E. coli* bacteria diluted in 1X Phosphate-buffered saline (PBS) buffer, 2 μL of 1X SYBR Gold, and 5 μL of 1X PBS buffer were loaded on top of their respective inlets and preloaded into the first valve. After the students ran their staining protocol from their remote device, the bacteria and dye were incubated in the central mixing valves for 20 min as per the SYBR Gold standard staining procedure. Once the staining period was complete, the stained bacteria were sequentially pushed to the optical detection region of the chip (Fig. 4B).

During this experiment, a high frame rate camera captured fluorescence events where stained *E. coli* bacteria were excited and emitted fluorescence signals (Fig. 4B). The resulting video is analyzed frame-by-frame, where the pixel intensities in the region of interest were integrated and plotted in the time domain. The resulting time domain fluorescence trace illustrated peaks in intensity if fluorescently stained bacteria were present. The time stamp associated with each event was also used to crop a 5 ms window around each event for visual confirmation of the detected event.

We evaluated the accuracy in completing the skeleton code that was given as homework. Successful groups correctly completed the skeleton code designed to fluorescently stain the *E. coli* bacteria samples. Based on this metric, 10 out of 11 participating groups (90.9%) successfully completed the skeleton code. The sole error was a minor fault that was easily rectified during the one-on-one session. Altogether, we generated an instructional module using IoT integrated LoC technology and implemented it in a context important to the student community in Bolivia. We conclude that this approach successfully exposed life sciences students to introductory concepts in computer programming.

### Lab-on-a-chip training leads to positive perspectives on computational science

Previously, it has been shown that experimental learning approaches in biology can increase the interest in pursuing STEM and continuing scientific careers^3,4,7^. We therefore aimed to understand whether using this module for teaching introductory programming concepts to life sciences students can also lead to positive feelings toward STEM. We therefore surveyed the students after completing the activity. Of note, because the surveys were not mandatory, 26 of the 34 students in the control group (76.5%), and 28 of the 42 students in the experimental group (66.6%) responded to the survey. Moreover, because we did not collect identifiable information from the students, we cannot match the answers from individual students. Therefore, we refrain from making paired comparisons in pre- and post-course surveys.

First, the students were asked to evaluate the theoretical and practical parts of the course. When asked how they would evaluate the theoretical part of the class, the large majority of respondents in both groups said that it was either excellent or good (92.3% control; 100% experimental) (*p* < 0.05) (Supplemental Fig. 5A). We then asked the respondents in the experimental group to evaluate the programming assignment. We found that 64.3% of the respondents said it was excellent, while 32.1% said it was good and 3.6% said it was regular (Supplemental Fig. 5B). Similarly, 82.1% of the respondents in the experimental group said that the programming project was useful to solidify concepts, while the rest felt neutral (Supplemental Fig. 5C). Notably, none of the respondents indicated that the programming coursework was not useful (Supplemental Fig. 5C). Additionally, all respondents in the experimental group said that the 1:1 call to receive feedback on their programming assignment was beneficial for their understanding of the concepts (Supplemental Fig. 5D). Because the students in the control group were not assigned a programming exercise, we did not ask them these questions (Supplemental Fig. 5B–D).

**Figure 5:**
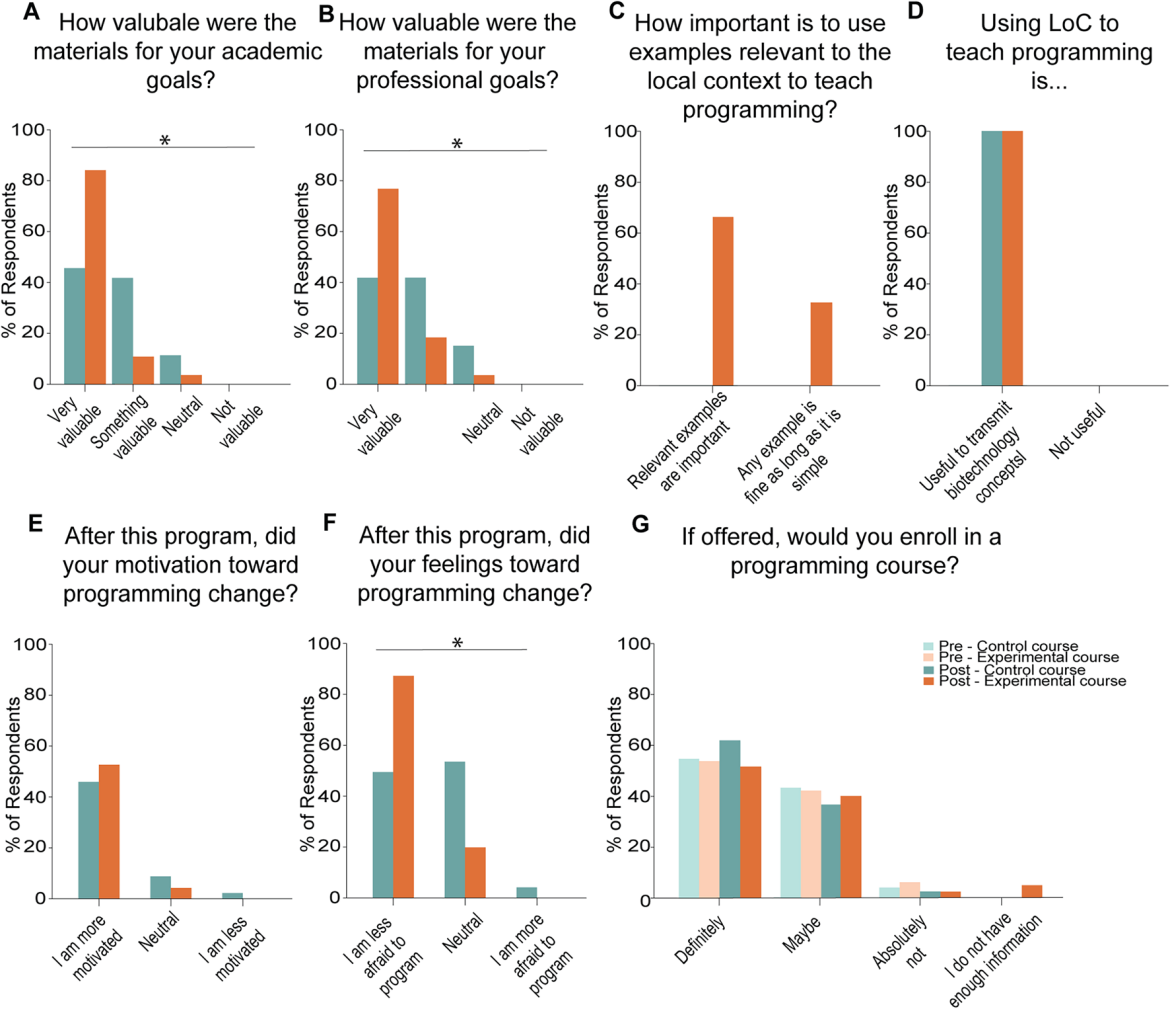
Program evaluation and prospects of careers in computational biology. (**A**–**G**) Program evaluation showing the distribution of students’ responses. (**A**) Perception of value of the program for academic goals. (**B**) Perception of value of the program for professional goals. (**C**) Importance of using context-aware projects in teaching. (**D**) Perceived applicability of LoCs in teaching programming. (**E**) Self-reported effects on motivation to learn to program. (**F**) Self-reported effects on feelings toward programming. (**G**) Comparison of intent to enroll in a programming course before and after this program. Control group: Pre-course n = 34; Post-course n = 26. Experimental group: Pre-course n = 42; Post-course n = 28. * = *p* < 0.05.

When asked whether they thought that the materials were valuable for their academic goals, 46.7% of the respondents in the control group and 85.7% of the respondents in the experimental group said the materials were very valuable, while 42.3% of respondents in the control group and 10.7% of respondents in the experimental group said it was somehow valuable (*p* < 0.05) (Fig. 5A). Similarly, 42.3% of respondents in the control group and 78.6% of the respondents in the experimental group said the materials were very valuable for their professional goals (*p* < 0.05) (Fig. 5B). Altogether, we conclude that students in the experimental group perceived a higher value of the activity than students that were not exposed to programming.

We then asked the students in the experimental group how comfortable they felt doing a programming-based assignment. We found that 96.4% of the respondents felt comfortable with the coding assignment (Supplemental Fig. 5E) suggesting that our exercise was effective in introducing programming concepts. Additionally, all respondents in the experimental group said that the project used was relevant to their degree (Supplemental Fig. 5F). We found that 64.3% of the respondents in the experimental group think that using examples relevant to the local context is important to teach programming (Fig. 5C). Strikingly, all respondents in both groups said that they thought that using LoC technologies to teach programming is a good example to transmit concepts in biotechnology (*p* > 0.05) (Fig. 5D), illustrating the applicability of this approach. In addition, the majority of respondents (80.0% control; 96.4% experimental) said that they would recommend this program to their peers (*p* > 0.05) (Supplemental Fig. 5G). Altogether, we observe that the students in the experimental group found the activity as appropriate for their background and to be useful for their degrees.

Given that the experimental group reported a high level of comfort in programming after the coursework, we then analyzed whether this comfort translated to more positive feelings toward programming, and how those feelings differed from the control group. We found that 80.8% of respondents in the control group and 92.9% of respondents in the experimental group felt more motivated to deepen their knowledge of programming (*p* > 0.05) (Fig. 5E). Strikingly, 82.1% of the respondents in the experimental group reported that they have less fear of programming after this activity (Fig. 5F). In contrast, only 46.2% of respondents in the control group reported less fear of programming, while 50% reported no changes after the activity (*p* < 0.05) (Fig. 5F). Interestingly, the overwhelming majority (96.2% control; 96.4% experimental) of the respondents thought that their degree should offer courses exclusively in computational biology (*p* > 0.05) (Supplemental Fig. 5H). In addition, we observed a high interest in taking computational biology classes with all respondents in the experimental group and 96.1% of respondents in the control group indicating that they would or at least would consider taking said classes (*p* > 0.05) (Fig. 5G). We conclude that a practical LoC-based activity reduces the fear of programming in biotechnology students.

**Figure 6:**
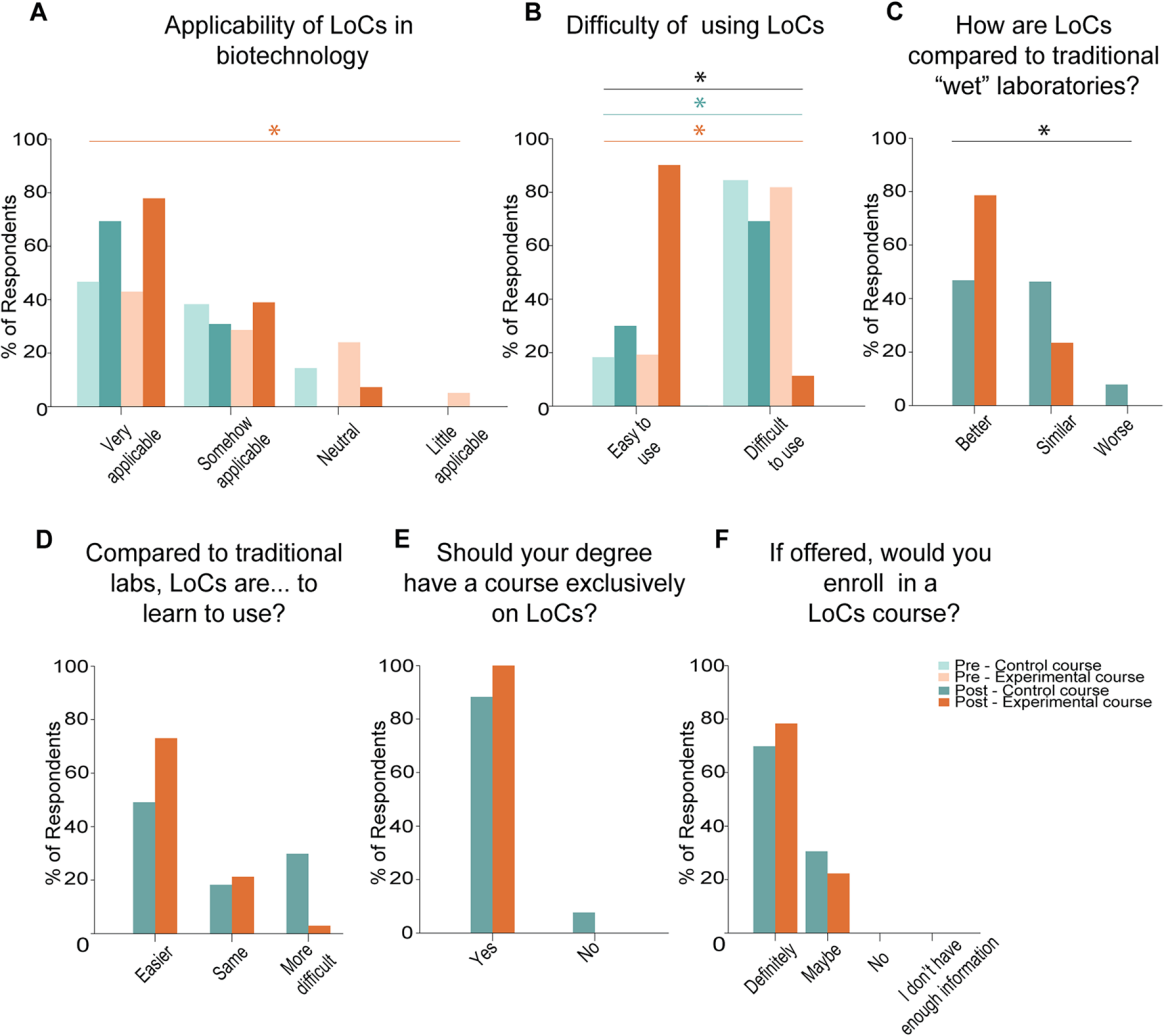
Context-aware experimental education led to a higher interest in LoCs. (**A**–**F**) Distribution of answers to pre- and post-course surveys. (**A**) Perceived applicability of LoC’s in biotechnology before and after the program. (**B**) Perceived difficulty of using LoCs before and after the program. (**C**) Perceived performance of LoCs as compared to traditional laboratories. (**D**) Perceived ease to learn to use of LoCs as compared to traditional laboratories. (**E**) Students’ interest in having a LoC-specific course in their degrees. (**F**) Self-reported interest to enroll in LoC-specific courses. Control group: Pre-course n = 34; Post-course n = 26. Experimental group: Pre-course n = 42; Post-course n = 28. * = *p* < 0.05. Black asterisk and bar = *p* < 0.05 is observed between control and experimental courses in the post-course surveys. Green asterisk and bar = *p* < 0.05 is observed between the pre and post-course surveys in the control course. Orange asterisk and bar = *p* < 0.05 is observed between the pre and post-course surveys in the experimental course.

Our survey then focused on questions regarding students’ interest in LoC technologies. We found that 96.2% of respondents in the control group and 100% of respondents in the experimental group reported that after our program they would want to learn more about LoC technologies (*p* > 0.05) (Supplemental Fig. 6A). We also observe that a high percentage of respondents find that LoCs are applicable to Biotechnology. Specifically, 69.2% of the respondents in the control group and 78.6% of the respondents in the experimental group thought that LoCs were highly applicable to biotechnology (*p* < 0.05 experimental pre vs. post course surveys; *p* > 0.05 control pre vs. post surveys; *p* > 0.05 control vs. experimental post course survey) (Fig. 6A). Remarkably, while 69.2% of respondents in the control group say that LoCs are easy to use after the course, the percentage is increased to 85.7% in the experimental group (*p* < 0.05 experimental pre vs. post course surveys; *p* < 0.05 control pre vs. post surveys; *p* < 0.05 control vs. experimental post course survey) (Fig. 6B). Altogether the results demonstrate that both courses lead to the perception that LoCs are easy to use, albeit at different levels. Furthermore, only the experimental course led to a perception of LoCs being applicable to biotechnology.

Next, we asked the students how they thought that LoCs performed compared to traditional molecular biology labs. We observed marked differences between the groups: 46.2% of the respondents in the control group found LoCs to be better than traditional labs, while another 46.2 thought that they were equal and 7.7% thought that they were worse (Fig. 6C). In the experimental group, we found that 78.6% of the respondents thought that LoCs were better than traditional labs, while the rest of the respondents thought they were equal (*p* < 0.05) (Fig. 6C). 50% of respondents in the control group and 75% of the respondents in the experimental group thought that LoCs were easier to learn to use than traditional labs, while 19.2% of respondents in the control group and 21.4% of them thought that the difficulty level was similar (*p* < 0.05) (Fig. 6D). Notably, 30.8% of respondents in the control group, but only 3.6% of respondents in the experimental group thought that LoCs were harder to learn than traditional labs (*p* < 0.05) (Fig. 6D). When we asked them what they thought were the primary advantages of LoCs, 50% of the respondents in the control group and 42.9% of the respondents in the experimental group thought that cost reduction was the main advantage (*p* > 0.05) (Supplemental Fig. 6B). All respondents in the experimental group and 92.3% of respondents in the control group thought that their degree should have a course exclusively on LoCs (*p* > 0.05) (Fig. 6E), and the majority of them (69.2% control; 78.6% experimental) said that they were sure they would take this course if it was offered (*p* > 0.05) (Fig. 6F). These results allow us to conclude that while both approaches (control and experimental) increase students’ interest in LoC technologies, the experimental approach leads to more comfort in the use of LoCs as replacement to traditional molecular biology labs.

**Figure 7:**
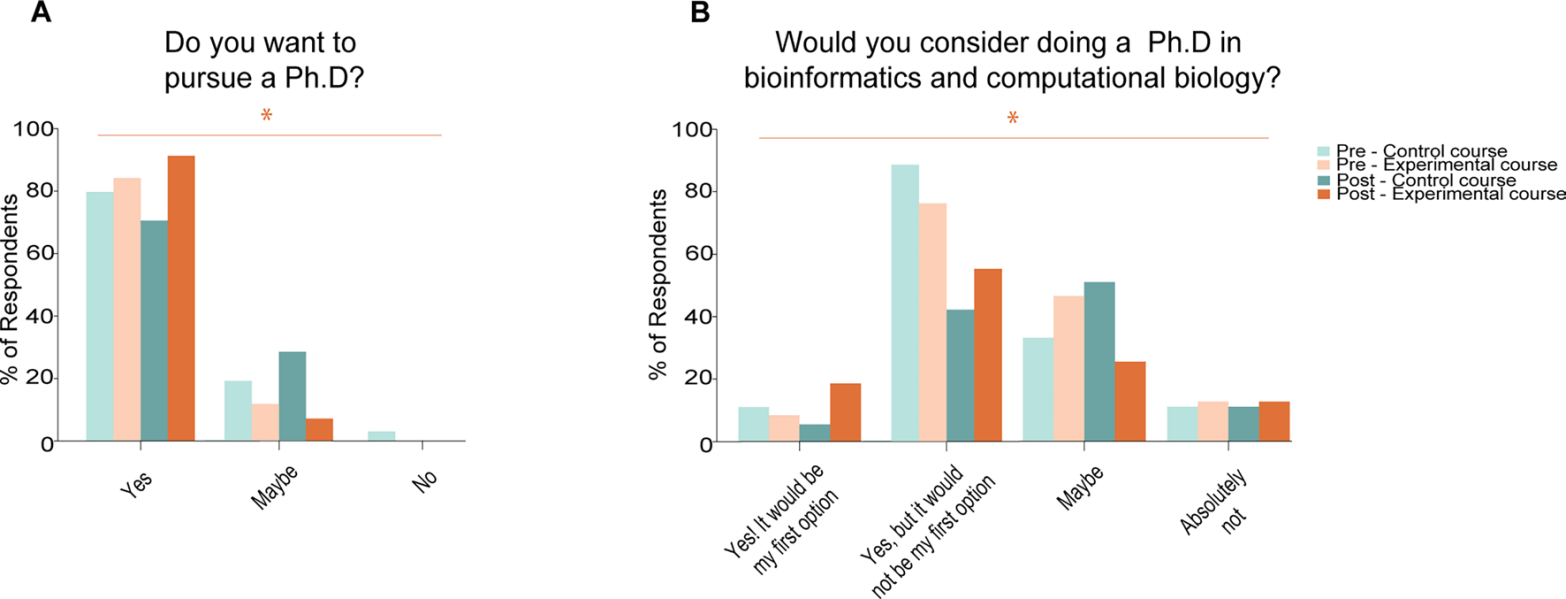
LoC-enabled context-aware experimental education leads to increased interest in pursuing PhDs. (**A**, **B**) Comparison of attitudes toward continuing PhD degrees before and after our programs. (**A**) Self-reported interest in pursuing doctoral studies. (**B**) Self-reported interest in pursuing doctoral studies in computational biology. Control group: Pre-course n = 34; Post-course n = 26. Experimental group: Pre-course n = 42; Post-course n = 28. * = *p* < 0.05. Orange asterisk and bar = *p* < 0.05 is observed between the pre and post-course surveys in the experimental course.

To understand the changes in the students’ outlook toward their careers, we then asked the students how important they thought programming was for a career in life sciences. In the control group, we found that 26.9% of the respondents thought that programming was very important, and 57.7% thought that it was important (Supplemental Fig. 7). On the other hand, we found that 57.1% of the respondents in the experimental group thought it was very important, while 35.7% thought it was important (*p* > 0.05 experimental pre vs. post course surveys; *p* > 0.05 control pre vs. post surveys; *p* > 0.05 control vs. experimental post course survey) (Supplemental Fig. 7). Remarkably, we observed a high percentage of respondents in both groups (80.8% control; 92.9% experimental) who want to pursue a doctorate after their undergraduate degrees (*p* < 0.05 experimental pre vs. post course surveys; *p* > 0.05 control pre vs. post surveys; *p* > 0.05 control vs. experimental post course survey) (Fig. 7A). Interestingly, after our course, most respondents in the experimental group (75%) said that they would consider doing a doctorate in computational biology, with 17.9% of them saying that it would be their first choice (Fig. 7B). In the control group, 69.2% of the respondents said they would consider doing a doctorate in computational biology, although only 7.7% said it would be their first choice (*p* < 0.05 experimental pre vs. post course surveys; *p* > 0.05 control pre vs. post surveys; *p* > 0.05 control vs. experimental post course survey) (Fig. 7B). Altogether, we conclude that both courses increase the perceived importance of programming among biotechnology students. However, only the experimental course increased the desire of students to continue their training toward a career in computational approaches to biology.

Finally, we asked the students if they had a message for the team. We found two themes among the respondents’ comments in the experimental group. One group of respondents demonstrated their excitement for their topic and their desire to learn more. Some quotes include:

- *“The classes were very interesting. I had never heard of this technology. After learning a little bit (about this technology), I want to study more programming. I feel (that learning programming) is something that would simplify many procedures.”*—Translated from Experimental Respondent 1.
- *“I wish you could share this project with many other young people who are interested in science. It is really impressive work.”*—Translated from Experimental Respondent 2.
- *“Here in Latin America, and especially in Bolivia, this type of technology does not exist, so I am very grateful for this experience.”*—Translated from Experimental Respondent 3.

The second group of respondents in the experimental group expressed their desire to get more involved in outreach initiatives related to programming and LoCs. Some example quotes include:

- *“It is a beautiful project. (I wish you) great success in improving your work and I hope that in the future courses you can accept volunteers from different countries to develop pilot programs or experiments.”*—Translated from Experimental Respondent 4.
- *“Thank you very much for giving us this opportunity and (I) wish you success in your present and future projects. Also, how can we get involved in more activities like these?”*—Translated from Experimental Respondent 5.
- *“We would like to get a little more involved in the development of the planning part.”*—Translated from Experimental Respondent 6.

In the control group, we found that students often mentioned that they wanted to learn more programming, although we did not find evidence of increased interest in getting more involved in LoC-based outreach activities:

- *“It is very important to understand new technologies, which is why I consider a programming course focused on biotechnology to be necessary.”*—Translated from Control Respondent 1.
- *“I would love to learn programming subjects. Although it is possible to learn programming online, having a space, a guide and especially extra time to learn would be incredible.”*—Translated from Control Respondent 2.

Altogether, we conclude that our approach designing a context-aware experimental learning module to expose life sciences students to introductory programming is effective at increasing their interest in the topic and LoC technologies, reduce their fear in programming, and increase their desire to continue graduate studies in computational biology.

## Discussion

Experimental learning has the potential to democratize STEM education, especially in the developing world^4,25,26^. Yet, it often requires resources, such as infrastructure, materials and supplies that are unattainable for most schools in remote regions^26^. Online learning, on the other hand, can scale STEM education with similar learning outcomes as in person training at a fraction of the cost^27^. Recent work has shown the ability to adapt several experimental modules to an either fully online^3,7^ or hybrid^28,29^ model taking advantage of IoT architectures^13^. Here, we extended this work by taking advantage of IoT-enabled LoCs to create projects that are tailored for a Latinx population in Bolivia. Previous work has applied similar approaches to computer programming training, although always in the context of sequence analysis^30,31^. Moreover, this work has been done with students with previous experience and interest in computational biology^30,31^. A novel aspect of our work was that we targeted Latinx students with little or no previous programming experience. We observed that the students not only increased their interest in learning to code, but also in continuing careers in computational biology.

By comparing the students who have undergone purely theoretical training and the students with both experimental and practical assignments, we uncovered several differences between the groups (Figs. 5, 6, and 7). Specifically, we observed that students in both groups had an increased interest in learning to program and continue careers with a programming focus. However, the students who had undergone the practical assignments reported less fear in programming and more comfort in using LoC technologies in the future. These results underscore the potential of internet-enabled LoCs for practical programming training.

An interesting aspect of the students’ feedback was that many participants in the experimental group asked to become volunteers to replicate and advance the project. Previous in person outreach activities in similar Latinx populations has shown this phenomenon where students often self organize into mentorship networks^25,32–34^. Future work, exploring the creation of communities of both mentors and mentees could be an important step toward solidifying this and other international outreach programs^35^. Moreover, the creation of online communities could help globalize the activities^36^ by creating transnational youth networks of students that transcend schools or countries to share information and learn^32,37^.

### Limitations of the study

A limitation of this study is that because the surveys were not mandatory only 26 of the 34 students in the control group and 28 of the 42 students in the experimental group responded to the post-course survey. Because we did not record any identifiable information from the students, we were unable to perform paired statistics comparing the changes in students’ behaviors and feelings before and after the course. Furthermore, we cannot discard the possibility that the students who answered the post-course survey were the ones who most enjoyed the course. Future studies that specifically focus on assessing the development of individual students will help us further understand the impact of this work. Moreover, comparing students in different geographical locations can help us expand our understanding on whether this approach would be universally beneficial or whether further modifications would be needed to target specific groups. For example, in our previous work where we used internet-enabled microscopy to complement existing courses in geographically Latinx populations, we unraveled several differences in STEM identity and desire to pursue STEM careers between Latinx students born in the United States and Latinx students in South America^3^.

In the present study, we used SYBR Gold dye to stain *E. coli* in otherwise pure water samples as a proxy for detection of contamination (Fig. 4B). However, this dye labels all DNA, including bacterial and eukaryotic DNA. We chose this dye due to its simplicity, as the staining does not require multiple incubations or secondary amplifications. More complex experiments capable of multiplexing could be beneficial to teach higher level courses. Indeed, we have previously developed similar LoC systems that can detect multiple pathogens in single samples by using either antibodies^38^or DNA probes^39^. The combination of more sophisticated probes and more complex experiments could become an important set of tools for computer programming instruction, particularly to students from a biology background.

Finally, the present study was performed in a relatively small number of students, and new iterations of the work in students from multiple backgrounds, locations and education levels are needed to truly understand the effects of remote LoC technologies in computer programming education. Here we included a tutorial for LoC design and assembly (Supplemental Notes 1 and 2), as well as the code that can enable others with expertise in LoC to replicate this study. In addition, we have recently established the UCSC Live Cell Biotechnology Discovery Lab as a platform for developing and expanding internet-enabled biology education. Through this center, we expect that new and interested users will collaborate in increasing the reach of these technologies to underserved populations worldwide.

### Conclusion

In conclusion, we developed context-aware experimental learning strategies to effectively target Latinx life sciences students and expose them to concepts in computer programming. By taking advantage of IoT-enabled LoCs we were able to create a curriculum on a topic of interest to the local community: testing of water quality. Using this approach in Bolivia, we showed that students gained knowledge to complete a relatively complex programming assignment and increased their interest in continuing training in computer programming applications to biology. We therefore propose that Internet-enabled experimental education can become a powerful tool to increase the representation of Latinx students continuing STEM careers and the STEM workforce.

## Methods

### Ethics statement

All methods were performed in accordance with the relevant guidelines and regulations. The University of California Santa Cruz Institutional Review Board reviewed and approved this work (HS-FY2023-42). The Ethics Committee at the Universidad Católica Boliviana San Pablo reviewed the protocol and approved it for execution in Bolivia (Protocol Approval 034). Informed consent was obtained from all participants that are part of this study.

### Participants

All participants were life sciences students at the Universidad Católica Boliviana San Pablo, based in the Santa Cruz de la Sierra campus. The students ranged from first to fourth year in college (18–22 years old). All participants self-identified as Latinx.

### Surveys

We surveyed students before and after the project using Google forms. We surveyed the students in Spanish. To more easily aggregate the data, most of the questions were presented either as Likert scale or multiple-choice questions. Specifically, the questions that assessed the students' feelings toward specific topics, such as programming and learning were done as Likert scales where students selected their level of agreement with specific statements. The questions assessed the previous experience of the student and their opinions about the barriers to the use of LoC technologies in Bolivia were presented as multiple-choice questions. At the end of the surveys we asked the students whether they had comments for the team or additional information that we should know. This question was presented as a short answer question. The surveys were anonymous and no identifiable information was collected. The surveys were not mandatory.

For the experimental group, a total of 42 students answered the pre activity survey (24% men and 76% women), while the post activity survey had 28 respondents (22% men and 78% women). For the control group, 34 students answered the pre activity survey (32% men and 68% women) and 26 answered the post activity survey (24% men and 76% women). The students were also given the opportunity to self-identify as non-binary.

### Statistics

All statistical analyses were done using R version 4.2.1 inside the RStudio environment. The data were first evaluated for normality using the Kolmogorov–Smirnov test. This test was selected to determine if the data deviated from a normal distribution, as it is suitable for comparing a sample with a reference probability distribution.

For comparisons between groups, the non-parametric Fisher's exact test was utilized. This test was chosen because it is particularly well-suited for categorical data with small sample sizes. The Fisher's exact test is a more accurate alternative to the chi-squared test when the expected frequencies are small, as it calculates the exact probability of observing the given data under the null hypothesis of no association between the variables. The Kolmogorov–Smirnov test was performed using the ks.test function, and Fisher's exact test was executed using the fisher.test function from the base R package. Statistical significance was set at *p* < 0.05 for all tests.

### Experimental course

The course consisted of 2 lectures of 1 h each. Both lectures were given in Spanish, the language of Bolivia. The first lecture was an introduction to LoC technologies and their applications to biotechnology, such as disease diagnosis and microbe detection. The students were given a short background on *Escherichia coli* infections and their impact in global health. The students were then introduced to the specific LoC device that they were going to use in the course, as well as the fabrication process for such device. In the second lecture, we introduced the students to the basic commands of the programming language used for controlling the LoC device. The students were run through simple exercises using the device. We went through each line of code to explain how the commands translated to operations in the device. To facilitate the understanding, we paired each line of code with an image of the LoC device which was filled with food coloring dyes to represent the liquid handlings.

After the 2 lessons, we gave the students the skeleton of a code to run a staining of *Escherichia coli* and detection of their presence in water samples. The students had 4 days to complete the assignment as groups of 3–4. They submitted their code through Dropbox.

Following the assignment, each group of students scheduled a 1-h long session with the engineers in charge of running the LoC platform. In these sessions, the students received feedback on their code and ran their code live. These sessions were in English, with simultaneous translation to Spanish. After running the code, the data was processed and sent back to the students.

### Control course

For the control, we did a single lecture of 1 h. The lecture was the exact same one as lecture 1 in the experimental group: it included an introduction to LoC and their use in biotechnology, biofabrication of LoC devices, as well as an introduction to *E. coli* and their relevance to global health. Because the control group lacked the programming exercise, we did not teach lecture 2.

### Chip design

In these experiments, we used a polydimethylsiloxane (PDMS) LoC device that combines sample preparation and optical detection functionalities. The design of our chip is based on the device published in^40^. Fifteen pneumatically controlled valves can be programmed to enable user-defined movement of nanoliter scale volumes of fluid through the device. Fabrication of this device followed standard photo and soft lithography and is described in^40^. We have included a step-by-step tutorial to create and assemble this chip as Supplemental Note 1. We note that the device fabrication steps are performed and calibrated at the University of California Santa Cruz clean room. Any individual and/or group with sufficient expertise and resources can fabricate a similar LoC platform following the fabrication steps. A simplified cost breakdown of fabricating one such PDMS LoC device in a similar clean room facility is also included as Supplemental Table 1. We also note that one of the major benefits of PDMS based soft lithography process is the design reusability that can easily bring down the fabrication cost. A custom-made electronic control box with digital I/O ports control the switching between positive and negative pressure to pneumatically actuate the PDMS valves. When negative pressure was applied to a valve, the PDMS lifted and allowed for fluidic exchange between adjacent channels within the valve volume. Positive pressure closed the valve and expels pushed liquid out of the valve and towards adjacent open valves. Six fluidic reservoirs are available for use as inlets and outlets. While three inlets were used in this instance, the ability to use all six inlets for an increased number of reagents shows the scalability of this experience to include even more complex microfluidic experiments and suit a wide array of outreach programs.

In this application, one inlet was used for loading *E. coli* bacteria (Thermo Fisher Scientific C404010), another inlet was used for loading 1 × SYBR Gold intercalating fluorescent dye, and the last inlet contained 1 × PBS buffer. After completing fluorescent staining of the *E. coli* bacteria, the sample was pushed through the device to an optical detection region. In this section of the chip, laser excitation light from an external, fiber-coupled 488 nm laser diode was coupled to a solid-core PDMS waveguide that orthogonally intersects the analyte channel. When a fluorescently stained bacterium flows through the excitation volume, it emits shifted fluorescence light that can be collected by a sensor. In this application, fluorescence signals from stained bacteria were collected by a high frame rate camera aligned above the optical detection region that had a 532 nm fluorescence filter in the optical path to remove any excitation light. The high frame rate of the camera ensured that the time resolution of the recorded video was also high and allowed for the detection of fluorescence events from individual particles.

### Integration of hardware to the cloud

The pneumatic control was obtained by connecting a stack of 3 port solenoid valves (SMC S070M-6DC-32) with the pneumatic inlets in the PDMS-based LoC device. The solenoid valve was connected with compressed air (positive pressure) and vacuum lines (negative pressure) and output either positive or negative pressure based on the input voltage level. An electronic relay (ULN2803 Switch Board) converted the logical voltage (0–3.3 V, 8 mA) level from the general purpose input/output (GPIO) pins of a programmable electronics to an appropriate voltage level (0–12 v, 1.5 A) for operating the solenoid valves. The electronic relay and solenoid valves are housed in a small box, called an “Automaton”. We used an internet-connected Raspberry Pi (Model 4) device as the programmable electronics in this instance. The PDMS LoC, solenoid valves, electronic relay, and Raspberry Pi construct the local IoT device. A step-by-step automaton electronics assembly guide is included as Supplemental Note 2 and a simplified cost breakdown of the local IoT device is included as Supplemental Table 2. The local IoT device was connected and controlled by a web-based graphical user interface (GUI) via Message Queuing Telemetry Transport (MQTT) protocol. The GUI acted as a virtual IoT device that could be easily accessed and operated from any remote location by any user with correct log-in information. The GUI was designed in web hosted Jupyter Notebook environment (wetAI) by a custom written Python program. A similar framework is previously described in^13^. The GUI offered three modes of operation. First, remote control of an individual pneumatic valve for manual operation was offered by a set of user-controlled buttons on the GUI. This mode helped to pre-wet the PDMS-based device, check functionalities of individual valves etc. However, this mode is not convenient for running a biological sample preparation step where one may need to sequentially turn on and off valves with pre-defined time delay. To fulfill this necessity, we developed a custom programming language with a predefined set of instructions and functions. A program written in this language is called script which is a text file that can be opened and edited in any text editor application. The structure of a script is very simple and intuitive, making it comprehensible for everyone irrespective of their educational background and programming related experience. Multiple scripts for common applications i.e., post-experiment device washing, can be saved in local IoT devices as templates for further use. The second mode is selection and execution of a locally saved script by the remote user for automatic sample preparation. Finally, a user can follow a template and write their own script for a particular application. The GUI also offers uploading a new script from a remote device and storing it locally for future usage. All these instructions are transferred as a MQTT message from the remote IoT device to the local IoT device placed in the laboratory. In the local IoT device, a Python-based interpreter program receives the MQTT message and decodes instructions as logic level information which is later output through GPIO pins.

### Interpreter program for decoding a “script”

We have developed a user-friendly custom programming language with predefined commands so that any user from different backgrounds can understand and write a program intuitively in this language. No prior knowledge about the GPIO pin orientation, logic level of Raspberry Pi hardware or expertise on conventional programming languages such as C/C++ or Python is required to operate our automaton. Additionally, our platform does not require any special application or integrated development environment (IDE) other than a text editor to be installed in the user side computer.

Similar to the C programming language, a “script” should have a “main” block with lines of instructions. Each instruction line starts with a command, defined by a special character or word. The current version of the program allows three single character commands, such as “o” for opening a valve, “c” for closing a valve and “w” for a wait period. “c” and “o” commands have the desired valve number and the “w” command has a wait period in milliseconds as a parameter. Also, additional user-defined functions can be called by writing “call” command followed by the function name. The definition of the user-defined function should be provided by the user after the “main” block. A simple flowchart of the interpreter program running in the background is demonstrated in Supplemental Fig. 8. We have included all the code to run this program in Github https://github.com/braingeneers/IOT_Education_Lab_on_Chip_Paper.

### Supplementary Information

Supplementary Information.

Supplementary Video 1.

## Data Availability

The informed consent given by the participants does not open for storage of data on an individual level in public repositories. Anonymized survey data are available upon request by qualified scientists. Requests require a concept paper describing the purpose of data access, ethical approval at the applicant’s institution, and provision for secure data access. Requests should be addressed to M.A.M-R. mmostajo@ucsc.edu.
