# Supplementary material for: Internet-enabled lab-on-a-chip technology for education
Source: Sci Rep. 2024 Jun 22;14:14364. doi: 10.1038/s41598-024-65346-0 (PMC11192768; doi:10.1038/s41598-024-65346-0)
Supplement: Supplementary file 1 — Supplementary Information. [file 41598_2024_65346_MOESM1_ESM.pdf]

**SCIENTIFIC REPORTS**
**SUPPLEMENTAL MATERIALS**

# Internet-enabled lab-on-a-chip technology for education

Tyler Sano*, Mohammad Julker Neyen Sampad*, Jesus Gonzalez-Ferrer, Sebastian Hernandez, Samira Vera-Choqqueccota, Paola A. Vargas, Roberto Urcuyo, Natalia Montellano Duran, Mircea Teodorescu, David Haussler, Holger Schmidt, Mohammed A. Mostajo-Radji

Supplemental Figure 1. Students' perceptions of a career in bioengineering and computational biology.
Supplemental Figure 2. Students' previous experience with the technologies used.
Supplemental Figure 3. Closing and opening pneumatic valves.
Supplemental Figure 4. Sample exercise for mixing E. coli and SYBR Gold in LoC.
Supplemental Figure 5. Program evaluation and prospects on similar courses.
Supplemental Figure 6. Context-aware experimental education led to high interest in LoCs.
Supplemental Figure 7. Perceived importance of programming in life sciences.
Supplemental Figure 8. Flowchart for the background interpreter program
Supplemental Note 1. Polydimethylsiloxane lab-on-chip device fabrication
Supplemental Note 2. Automaton electronics assembly guide
Supplemental Table 1. Cost breakdown for polydimethylsiloxane lab-on-chip device fabrication
Supplemental Table 2. Cost breakdown for local IoT device
Supplemental Video 1. Video demonstration of mixing food dye

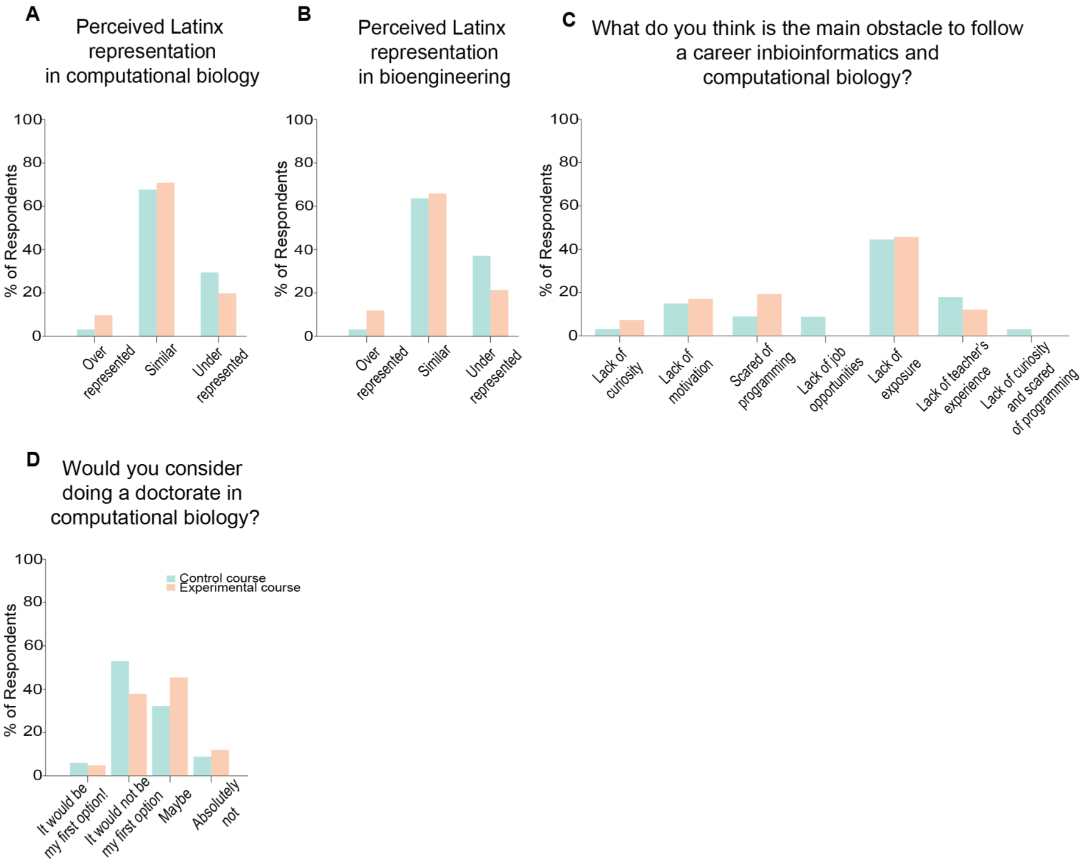


**Supplemental Figure 1. Students' perceptions of a career in bioengineering and computational biology.** (A-D). Distribution of answers to pre-course survey questions. A) Perceived representation of Latinx in computational biology. B) Perceived representation of Latinx in bioengineering. B) Perceived obstacles of following a career in computational biology. C) Perceived obstacles of following a career in computational biology. D) Desire to perform a doctorate in computational biology. n = 34 students in the control group and 42 students in the experimental group.

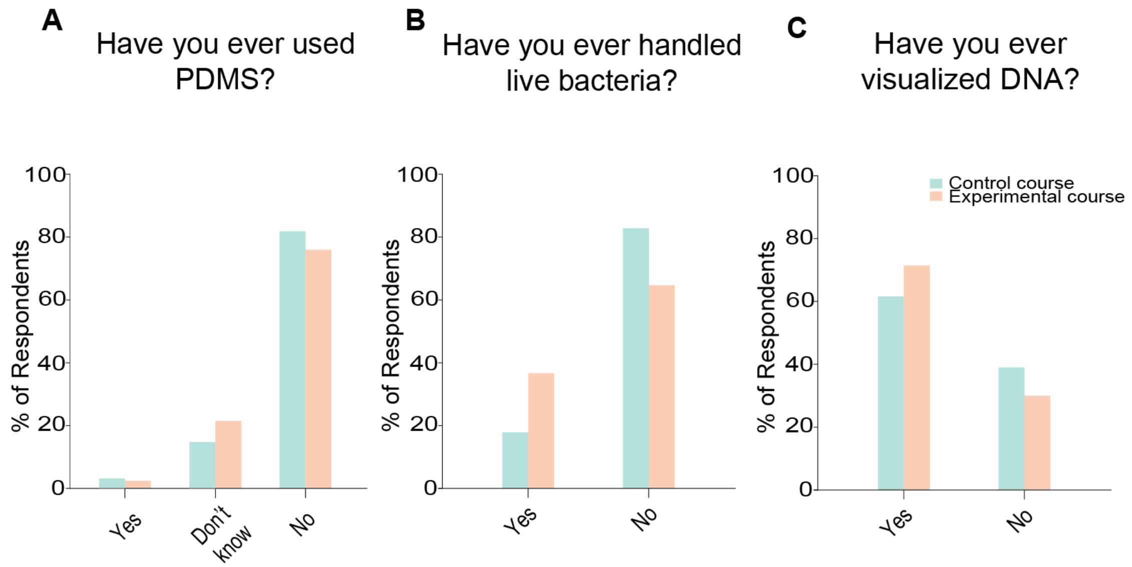


**Supplemental Figure 2. Students' previous experience with the technologies used.** (A-C). Distribution of answers to pre-course survey questions. A) Previous experience using PDMS. B) Previous experience handling bacteria. C) Previous experience visualizing DNA. n = 34 students in the control group and 42 students in the experimental group. * = $p<0.05$.

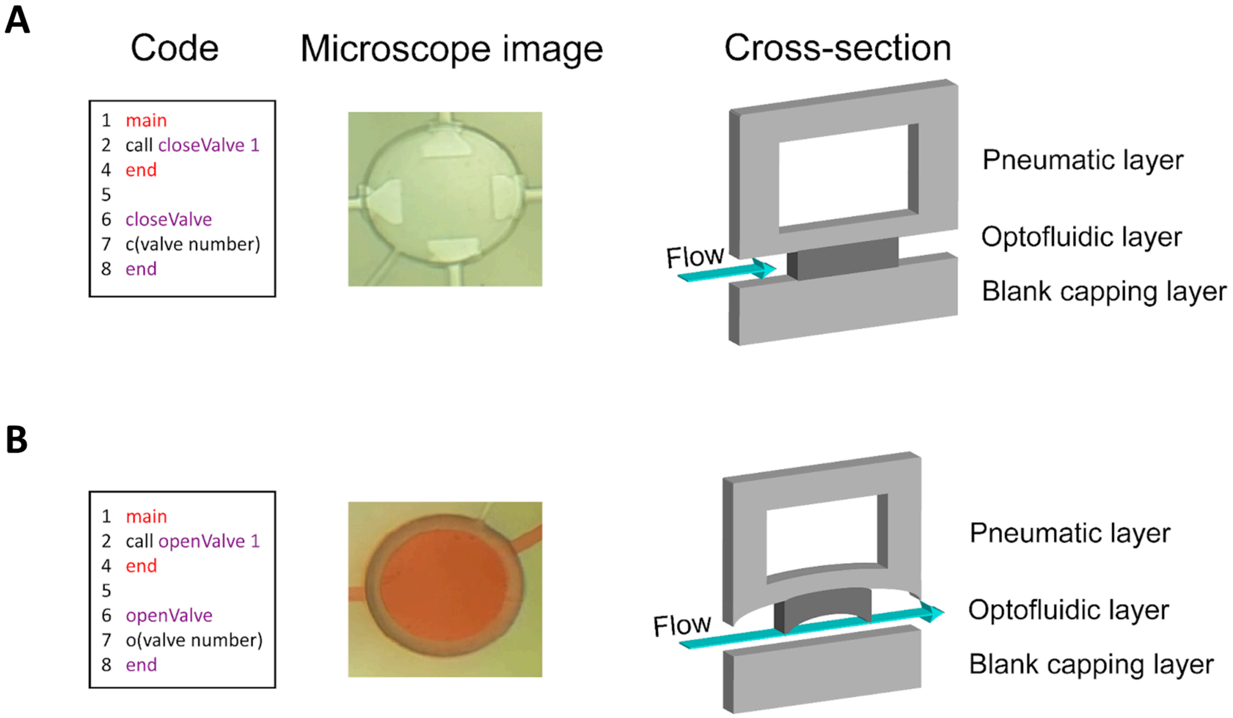


**Supplemental Figure 3. Closing and opening pneumatic valves.** a) Demonstration of the code used to close a specified valve. The microscope image illustrates what the valve looks like from the top when closed, while the cross-section demonstrates how flow is stopped. b) Demonstration of the code used to open a valve with the corresponding microscope image of a valve opened and filled with red food dye. The cross-section diagram illustrates how flow is enabled when the valve is opened.

```
main
call rotateCW ??
call wait 1
end

rotateCW
o??
call wait 1
c??
call wait 1
o??
call wait 1
c??
call wait 1
o??
call wait 1
c??
call wait 1
o??
call wait 1
c??
end

wait
w1000
end
```

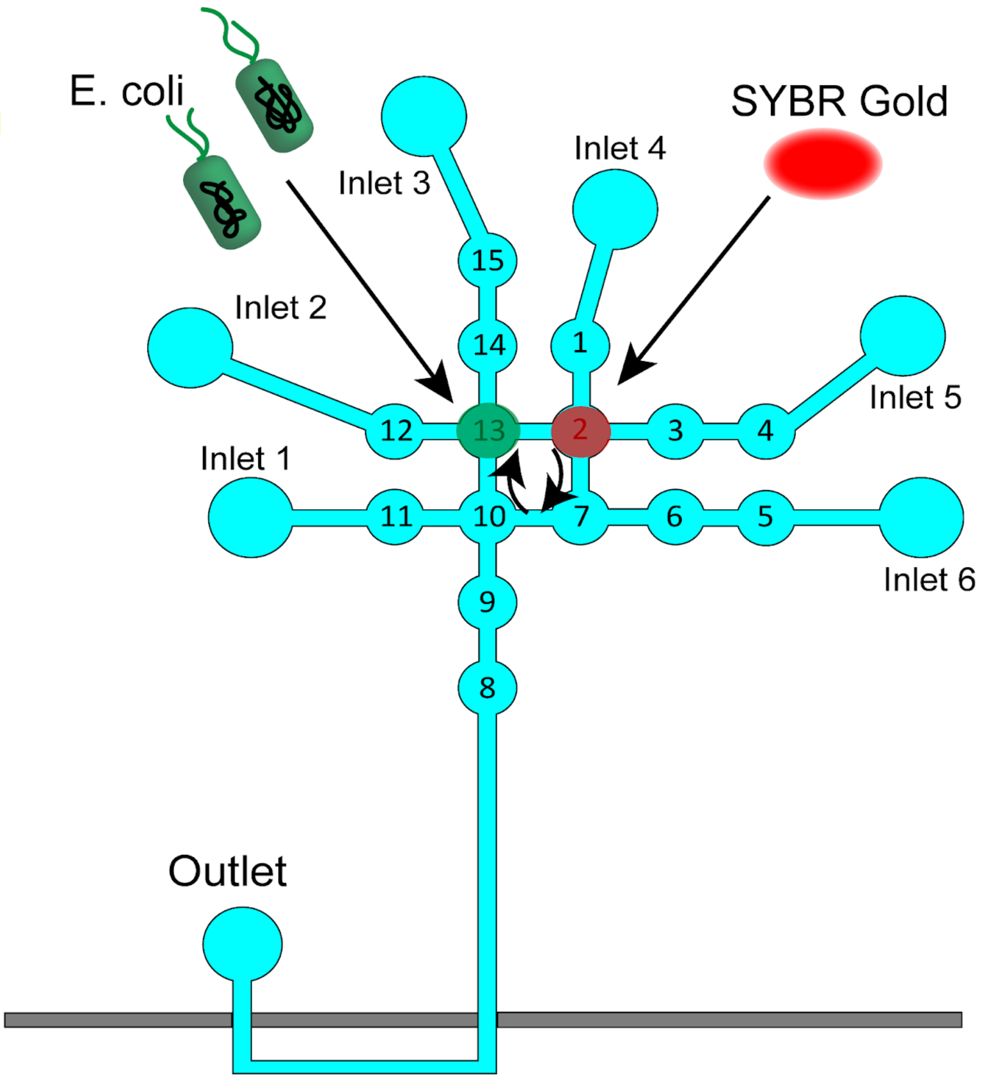


**Supplemental Figure 4. Sample exercise for mixing E. coli and SYBR Gold in LoC.** This figure illustrates an example of an assignment given to students asking them to fill in a redacted code. Students are given information that sample volumes of E. coli and SYBR gold are located in valves 13 and 2, respectively, and asked to fill in the valve numbers to mix the sample volumes in the clockwise direction for a given number of cycles.

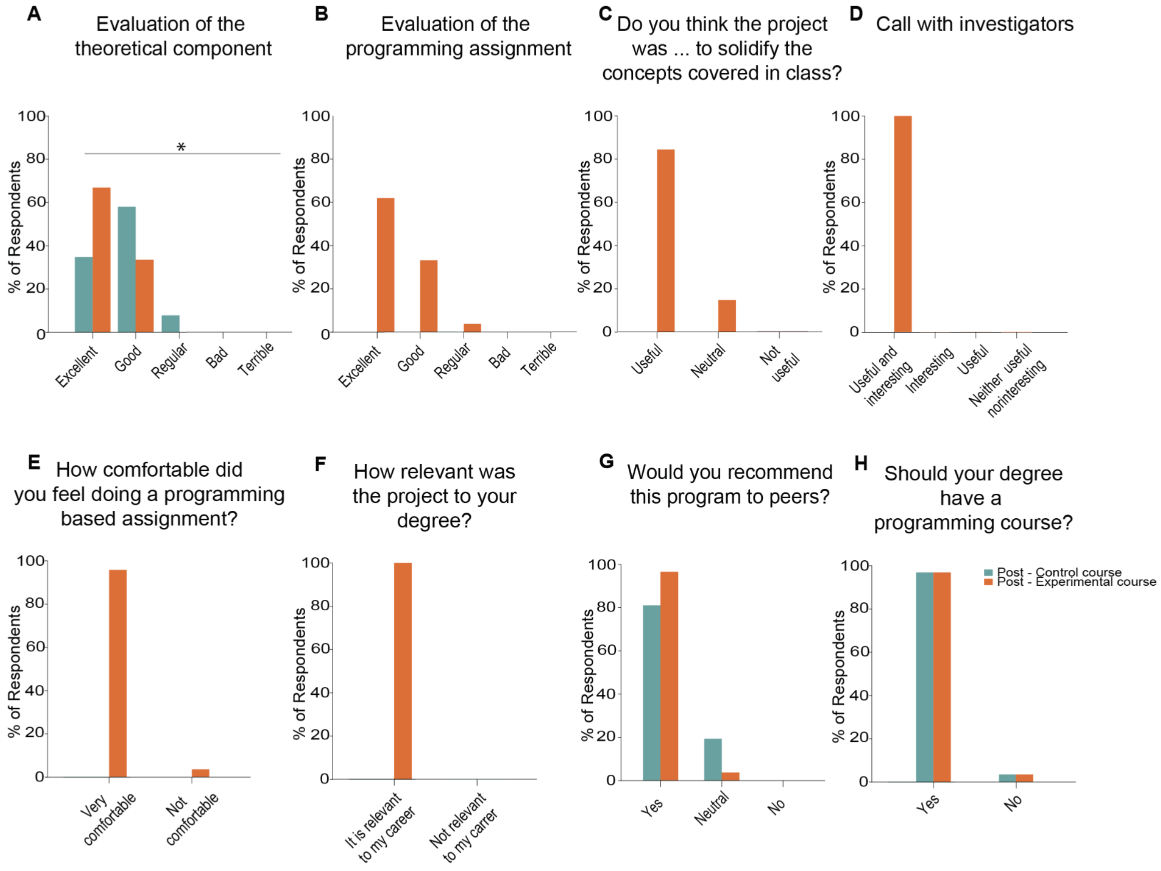


**Supplemental Figure 5. Program evaluation and prospects on similar courses.** (A-H) Program evaluation showing the distribution of students' responses. A) Evaluation of the theoretical component of the program. B) Evaluation of the programming assignment. C) Perception of the usefulness of the programming assignment to solidify concepts. D) Perception of the usefulness of the call with investigators to receive feedback on programming assignments. E) Self-reported comfort in completing the programming assignment. F) Perceived relevance to the programming assignment to the students' degrees. G) Students' recommendations to their peers. H) Student's perceptions on whether their degree should have a programming course. Control group post-course n = 26. Experimental group post-course n = 28. * = $p<0.05$.

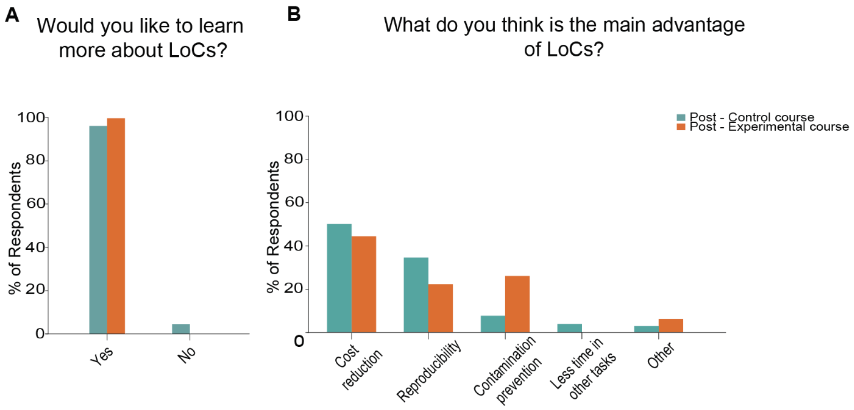


**Supplemental Figure 6. Context-aware experimental education led to high interest in LoCs.** (A-B). Distribution of answers to post-course survey questions. A) Self-reported interest in learning more about LoCs. B) Perceived main advantage of LoCs in biotechnology. Control group: n = 26. Experimental group: n = 28.

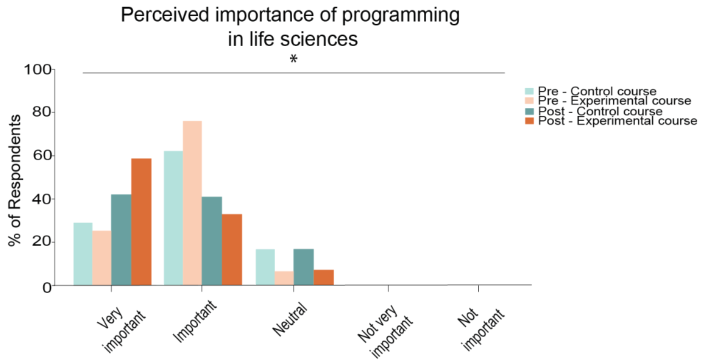


**Supplemental Figure 7. Perceived importance of programming in life sciences.** Control group: Pre-course n = 34; Post-course n = 26. Experimental group: Pre-course n = 42;. Post-course n = 28. * = $p<0.05$. Black asterisk and bar = $p<0.05$ is observed between control and experimental courses in the post-course surveys.

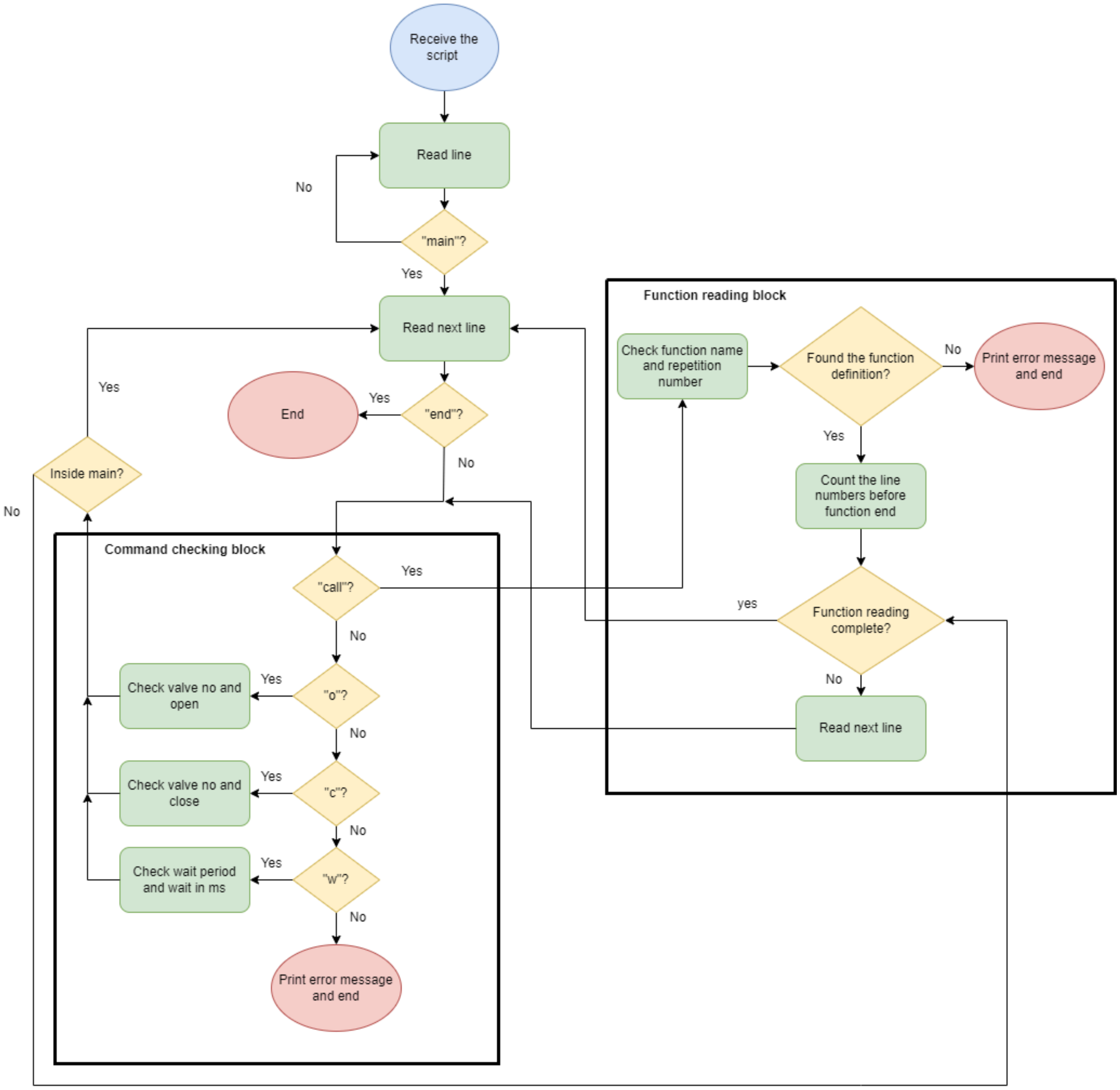


**Supplemental Figure 8. Flowchart for the background interpreter program.** Details of background program for interpreting a "Script". This flowchart visually illustrates the receiving of the script that controls the automaton. When the main function is found, the interpreter program is able to read commands and parse other functions in the text file to sequentially open and close the valves as well as run user defined functions.

## Supplementary Note 1: Polydimethylsiloxane lab-on-chip device fabrication

Table of Contents

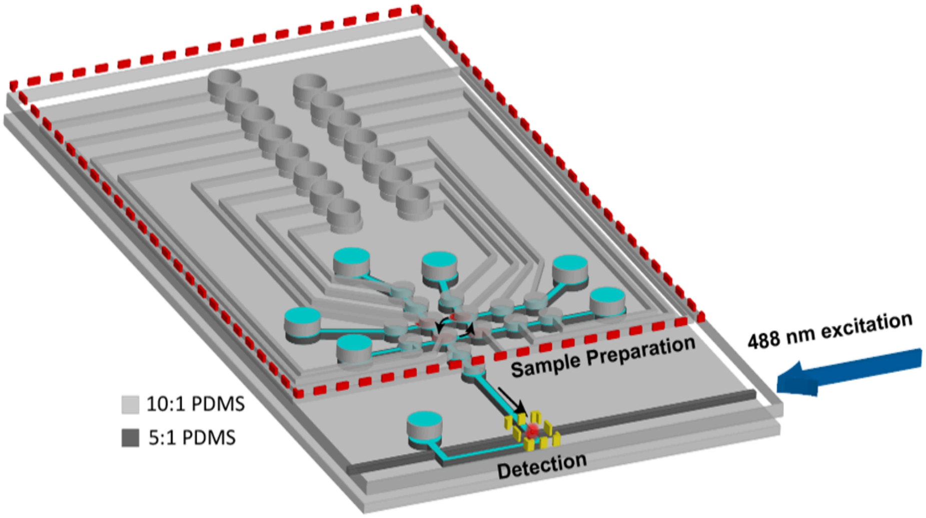


List of components........................................................................................................ 5
Fabrication Guide........................................................................................................ 5
1. Mask Preparation........................................................................................ 5
2. SU-8 master fabrication.............................................................................. 6
3. PDMS fabrication........................................................................................ 9

**List of components**

1. List of chemicals
    a. Isopropyl alcohol (IPA)
    b. Acetone
    c. SU-8 2005
    d. SU-8 developer
    e. Sylgard 184 elastomer (base and curing agent)
    f. Silane
2. List of instruments
    a. Heratherm oven - Thermo Scientific.
    b. Reactive Ion Etcher - Plasma Equipment.
    c. Digital hot plates - HP-51, Torry Pines Scientific Inc.
    d. Spin coater - Model PWM50, Headway Research Inc.
    e. Karl Suss mask aligner - Surplus Process Equipment Corp.
    f. OAI 204IR Mask Aligner - Optical Associates Inc.
    g. Desiccator - SP Bel-Art
3. Miscellaneous
    a. 30 gauge needle & syringe - BD
    b. 1 mm punch – Integra Miltex
    c. Plastic cup – 8 oz capacity
    d. Custom made circular acrylic mold.
4. Software
    a. AutoCAD (available: https://www.autodesk.com/)

**Fabrication Guide**

The complete PDMS LOC device fabrication can be divided into 3 steps. The fabrication detail is collected from T. Sano et.al., Biosensors 2022, 12(7), 501; https://doi.org/10.3390/bios12070501.

***1. Mask Preparation***

In general, microfluidic features are conventionally transcribed using CAD software. The designs in this study were created using AutoCAD as this commercial software is free to students. The design is divided into two layers (see Figure SN 1a). The fluidic and waveguide mask is drawn in purple color (Figure SN 1b) and the pneumatic control mask is drawn in yellow color (Figure SN 1c). Usually, the design is scaled with respect to a 100mm diameter silicon wafer so multiple devices can fit inside a single design.

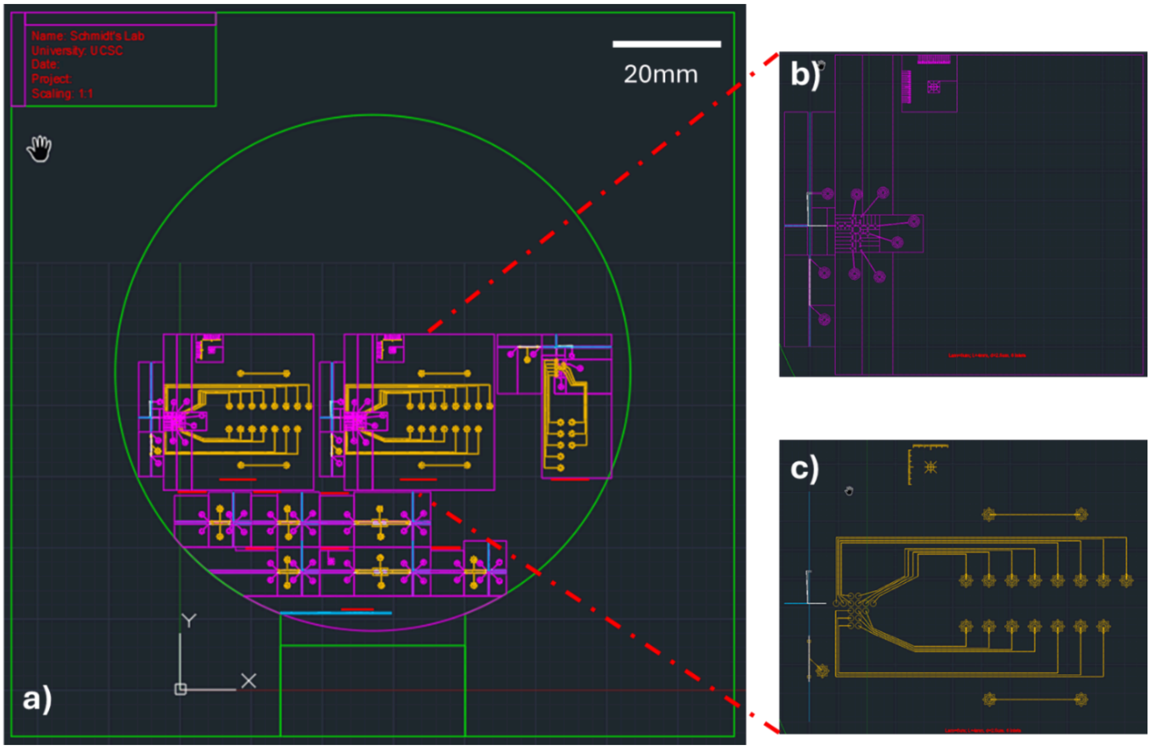


**Figure SN1**: Mask design in AutoCAD.

Once the features have been defined digitally, they are printed either on a Mylar transparency— when feature size limits don't shrink below 10µm—or are laser printed on a chrome mask with ~1µm precision. For the design used in this study, pneumatic layer mask was printed in Mylar transparency mask (Artnet Pro) and the waveguide & fluidic layer was printed in chrome mask (HTA photomask, San Jose, CA).

### 2. *SU-8 master fabrication*

The master fabrication utilizes standard SU-8 lithography procedure to transfer designs from the photomask to the SU-8 coated silicon wafer. These master wafers will be later used for molding PDMS structures. The steps may vary from cleanroom to cleanroom. A general fabrication workflow for the University of California Santa Cruz cleanroom is presented below.

1) Silicon Wafer Preparation
    a) Take two blank silicon wafers. One for the waveguide & fluidic layer and another for the pneumatic control layer.
    b) Spray blank Si wafer with IPA, blow dry with Nitrogen.
    c) Bake in 60°C oven for more than 1 hour (overnight if possible).
    d) Reactive ion etch in oxygen plasma at 80% power for 10 min.
    e) Cover spin coater with aluminum foil.
    f) Load wafer onto spin coater.

For waveguide & fluidic layer:

2) SU8 2005 Spin procedure
    a) Carefully pour SU-8 2005 onto the wafer until about 2/3 of the wafer is covered.
    b) Use the following spin procedure programmed into spin coater.
    c) While the wafer is spinning, program hotplates to 65°C and 95°C.

| Step | Speed (RPM) | Time (sec) | Ramp (RPM/sec) |
|---|---|---|---|
| 1 | 500 | 10 | 100 |
| 2 | 3500 | 60 | 300 |
| 3 | 0 | 0 | 500 |

3) Follow SU-8 2005 pre-exposure bake protocol outlined below:

| Step | Temperature (°C) | Time (min) |
|---|---|---|
| 1 | 65 | 1 |
| 2 | 95 | 6 |
| 3 | 65 | 1 |
| 4 | 25 | 3 |

4) Edge Bead Removal
    a) Conduct edge bead removal using acetone loaded into a syringe with a small gauge needle (30 gauge).
    b) Use the following spin procedure programmed into spin coater.
    c) While the wafer is spinning at 700 RPM, remove ~2-3 mm of SU-8 from the outside edge of the wafer. The needle should be angled at 30° with respect to the horizontal axis. Continue spraying until the edge bead is removed and slowly move stream off of the wafer.

| Step | Speed (RPM) | Time (sec) | Ramp (RPM/sec) |
|---|---|---|---|
| 1 | 700 | 30 | 100 |
| 2 | 1500 | 5 | 500 |
| 3 | 2500 | 10 | 1000 |
| 4 | 0 | 0 | 500 |

5) UV Exposure
    a) Follow SOP for Karl Suss mask aligner to turn on and then load the mask.
    b) Measure output flux from UV lamp using power meter.
    c) Load and align SU-8 coated wafer and bring wafer into contact with the waveguide & fluidic mask.

d) Ensure “Hard Contact” is enabled and then expose wafer with total dosage of 69.8 $mJ/cm^2$.
e) Follow post-exposure bake protocol outlined below:

| Step | Temperature (°C) | Time (min) |
|---|---|---|
| 1 | 65 | 6 |
| 2 | 95 | 6 |
| 3 | 65 | 7 |
| 4 | 25 | 10 |

6) Development
   a) Alternate between approximately 8 seconds of SU-8 developer and wash with IPA to prevent washing away any features.
   b) Check features under microscope to ensure fully developed Repeat this process until all residue is removed while being careful not to lift off features.
7) Hard Bake
   a) Hard bake @ 200°C for 5 minutes to reflow SU-8.
   b) Ramp wafer to 65°C first for 5 minutes- this can help with cracked features.

For pneumatic control layer:

8) SU8 2005 Spin procedure
   a) Carefully pour SU-8 2005 onto another wafer until about 2/3 of the wafer is covered.
   b) Use the following spin procedure programmed into spin coater.

| Step | Speed (RPM) | Time (sec) | Ramp (RPM/sec) |
|---|---|---|---|
| 1 | 500 | 10 | 100 |
| 2 | 3000 | 120 | 300 |
| 3 | 0 | 0 | 500 |

9) Follow SU-8 2005 pre-exposure bake protocol outlined below:

| Step | Temperature (°C) | Time (min) |
|---|---|---|
| 1 | 65 | 5 |
| 2 | 95 | 30 |
| 3 | 65 | 5 |
| 4 | 25 | 5 |

10) Edge Bead Removal
   a) Same as step- 4

11) Exposure

c) Load the pneumatic control layer mask into Karl Suss mask aligner.
d) Load and align SU-8 coated wafer and bring wafer into contact with the mask.
e) Ensure “Hard Contact” is enabled and then expose wafer with total dosage of 146.3 mJ/cm$^2$.
f) Follow post-exposure bake protocol outlined below:

| Step | Temperature (°C) | Time (min) |
|---|---|---|
| 1 | 65 | 5 |
| 2 | 95 | 15 |
| 3 | 65 | 10 |
| 4 | 25 | 10 |

12) Development

a) Alternate between approximately 30 seconds of SU-8 developer and wash with IPA to prevent washing away any features.
b) Check features under microscope to ensure fully developed Repeat this process until all residue is removed while being careful not to lift off features.

13) Hard Bake

a) Hard bake @ 200°C for 5 minutes to reflow SU-8
b) Ramp wafer to 65°C first for 5 minutes- this can help with cracked features.

### *3. PDMS fabrication*

The design transfer to the PDMS is done through replica molding, a soft lithography process by casting the liquid pre-polymer of an elastomer over a master that has patterned structures on its surface. The liquid form is prepared by mixing Sylgard 184 elastomer (consists of base and curing agent) at a predefined ratio. For a thicker layer (~millimeter), the liquid is directly poured within a cast, and for a thinner layer (~micrometer) the liquid is coated by a spin coater. The details are provided below.

1) PDMS mixing

a) Mix 12 grams of 5:1 PDMS and two cups of 55 grams of 10:1 PDMS.
b) For devices with a pneumatic waver, mix an additional cup of 11 grams of 10:1 PDMS.
c) Degas PDMS for 2 hours in the PDMS desiccator.

2) Waveguide layer fabrication

a) Ensure waveguide & fluidic master wafers is clean- Wash with IPA and blow dry if necessary.
b) Silanize the wafer for 40 minutes in silane desiccator using 3-4 drops of silane.

c) Keep wafer as far away from silane source as possible to keep wafers clean.
d) Blow dry wafer after silane treatment to remove any particles from the surface.
e) Use the following spin procedure for spinning 5:1 PDMS.
f) Cure 5:1 PDMS-coated wafer in oven (60°C) for 2 hours. (see Figure SN 2 left panel)

| Step | Speed (RPM) | Time (sec) | Ramp (RPM/sec) |
|---|---|---|---|
| 1 | 500 | 10 | 100 |
| 2 | 5000 | 900 | 600 |
| 3 | 0 | 0 | 500 |

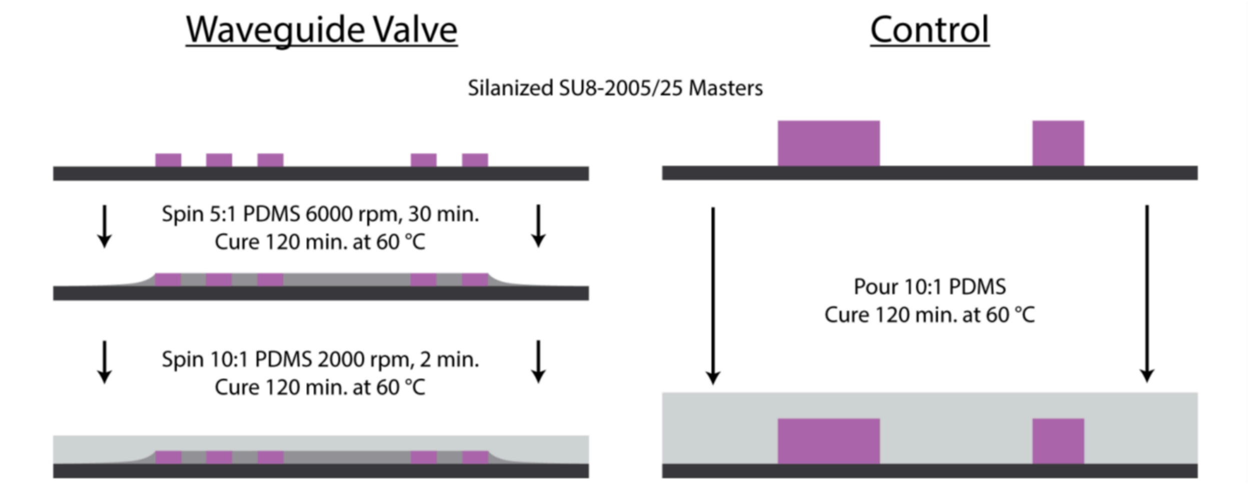


**Figure SN2: Design transfer to PDMS layers workflow.** a) thin layer of PDMS fabrication using spin coater and b) thicker layer of PDMS fabrication using circular acrylic mold.

2) Membrane fabrication
   a) Load 5:1 PDMS-coated wafer back into spin coater and spin the 11 g of 10:1 PDMS using the following program:

| Step | Speed (RPM) | Time (sec) | Ramp (RPM/sec) |
|---|---|---|---|
| 1 | 500 | 10 | 100 |
| 2 | 1000 | 300 | 500 |
| 3 | 0 | 0 | 500 |

3) Pneumatic control layer fabrication
   a) Clean and salinize the pneumatic control master wafers as described in the waveguide layer fabrication.

   b) load the master wafer into thin (3mm), circular acrylic molds.
   c) Pour 10:1 PDMS on the wafer close to surface to prevent bubbles (see Figure SN 2 right panel).
   d) Carefully place acrylic plate with polygons over the top of pneumatic layer without creating bubbles and then secure with screws.
4) Blank substrate layer fabrication
   a) Take a blank silicon wafer. Then clean and salinize the wafer following the same procedure as the waveguide layer.
   b) Pour 10:1 PDMS on the acrylic mold (3mm) bound blank wafer as described in the pneumatic control layer fabrication.
5) Cure all three layers, waveguide layer with membrane, pneumatic control layer, and the blank layer in 60°C oven for 2 hours (overnight if possible)
6) Pneumatic control layer bonding with waveguide layer
   a) Remove acrylic backing, silicon pneumatic master, and circular mold carefully using IPA.
   b) PDMS pneumatic layer should remain in contact with acrylic plate with polygons.
   c) Load pneumatic layer and waveguide layer into OAI mask aligner.
   d) Use substrate vacuum to keep waveguide wafer in place.
   e) Roughly align and mark placement of wafer and mask.
   f) Load both layers into RIE and plasma treat at 20% of the power for 30 seconds.
   g) Place pneumatic layer and waveguide wafer back into OAI mask aligner.
   h) Realign and then carefully bring the waveguide wafer into contact with pneumatic layer.
   i) Turn off substrate vacuum and remove bonded layers from mask aligner and cure in oven for 2 hours (overnight if possible). (see Figure SN 3a)
7) Substrate bonding
   a) Separate acrylic polygon plate and silicon waveguide master and tape exposed waveguide surface.
   b) Mark fluidic and pneumatic inlets/outlets using a sharpie and punch using a 1 mm biopsy punch.
   c) Separate blank layer from silicon wafer.
   d) cut chips individually and cut identical shapes out of blank layer.
   e) Remove tape and treat each chip and corresponding blank with O2 plasma in RIE at 20% of the power for 30 seconds.
   f) Place the top half of chip on vacuum line such that vacuum is only applied to pneumatic inlets – this procedure is followed to prevent the irreversible bonding of the microfluidic valve with the blank substrate layer.

g) Carefully overlay blank on exposed surface and bond chip together. (see

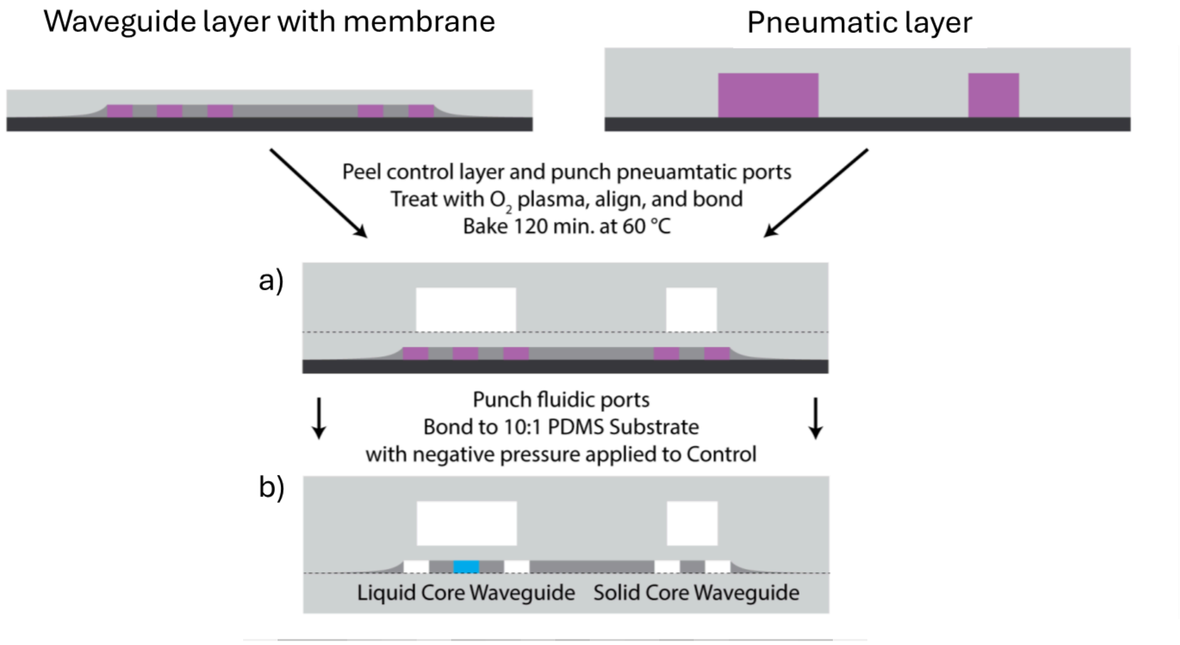


**Figure SN3. PDMS layers bonding workflow.**

## Supplementary Note 2: Automaton electronics assembly guide

Table of Contents

List of components........................................................................................ 13

Assembly Guide........................................................................................... 13

1. Electronic assembly................................................................................. 13
2. Tubing assembly...................................................................................... 16

List of components

1. List of electronics
    a. 12V DC adapter
    b. 3 port Solenoid valve (SMC S070M-6DC-32) x16
    c. Raspberry Pi 3B+ or higher version - with power supply & SD card
    d. Female-to-female jumper wire x20
2. List of tubing & assembly
    a. Air and vacuum supply port (SS070M01–2A, SS070M01–3A)
    b. Gaskate (SS070M-80A-1) x16
    c. Clip (SS070M-80A-2) x16
    d. ø3.18/ø2 tube for valves
    e. ø6/ø4 tube for vacuum and air application
    f. Male Luer to Hose Barb Fittings x16
    g. Stainless steel dispensing needle (1mm) x16
    h. Stainless steel dispensing bent needle (1mm) x16
    i. 1/32” inner diameter tube
    j. Nut and bolt (ø3.4) x10

**Assembly Guide**

The complete automaton electronics assembly can be divided into 2 steps.

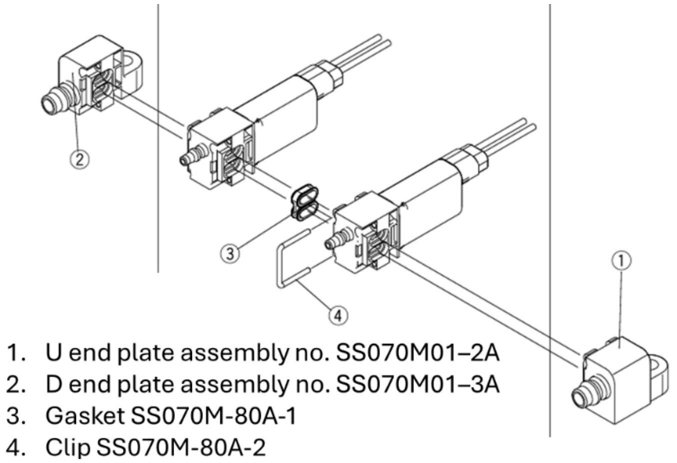


***SN4. Solenoid valve assembly.*** *Adapted from [https://www.smcpneumatics.com/pdfs/S070.pdf](https://www.smcpneumatics.com/pdfs/S070.pdf)*

***4. Electronic assembly***

a) First an array of 16 solenoid valves is assembled with D and U end terminating plates for air and vacuum applications respectively (See Figure SN 4).

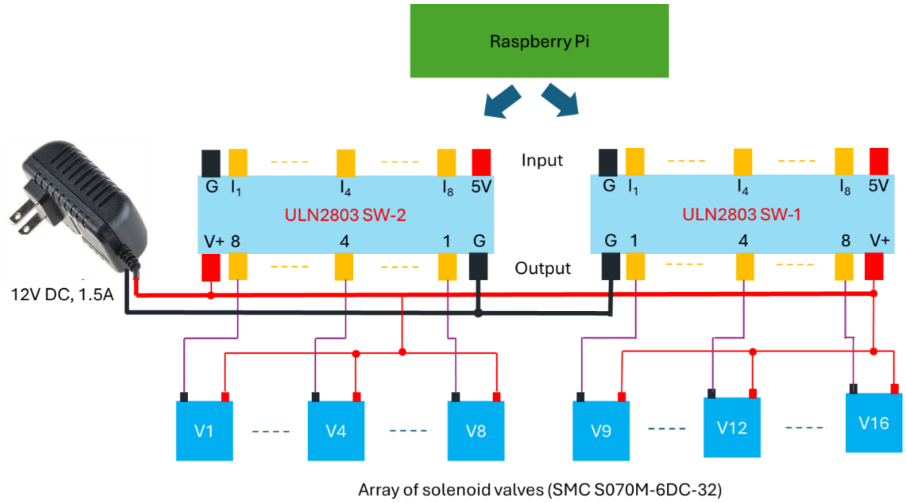


**Figure SN 5: Circuit diagram for the automaton electronics box.**

b) Use nut and bolt to fix relay switch board and the valve array to a cardboard.
c) Use electric jumper cables to complete the circuit following the pin diagram shown in Figure SN5-6.

| Relay switch input | Raspbery Pi GPIO |
|---|---|
| SW-2 : 5V | 2 |
| SW-1 : 5V | 4 |
| SW-2 : G | 39 |
| SW-1 : G | 6 |

| Solenoid Valve | Relay switch input | Raspbery Pi GPIO |
|---|---|---|
| 1 | SW-2 : 8 | 11 |
| 2 | SW-2 : 7 | 13 |
| 3 | SW-2 : 6 | 15 |
| 4 | SW-2 : 5 | 16 |
| 5 | SW-2 : 4 | 18 |
| 6 | SW-2 : 3 | 12 |
| 7 | SW-2 : 2 | 22 |
| 8 | SW-2 : 1 | 29 |
| 9 | SW-1 : 1 | 40 |
| 10 | SW-1 : 2 | 38 |
| 11 | SW-1 : 3 | 36 |
| 12 | SW-1 : 4 | 32 |
| 13 | SW-1 : 5 | 37 |
| 14 | SW-1 : 6 | 35 |
| 15 | SW-1 : 7 | 33 |
| 16 | SW-1 : 8 | 31 |

**Figure SN 6: Pin diagram for Raspberry Pi - relay switch- solenoid valve connection.**

## *5. Tubing assembly*

a) Use the ø6/ø4 tube for vacuum and air application.
b) Cut the ø3.18/ø2 tube at suitable length to connect individual valves with male luer to hose barb fittings.
c) Connect 1mm inner diameter bent needle to one end and the blunt straight needle with luer lock to the other end of a 1/32” inner diameter tube.
d) Connect the luer lock needle with the male luer fitting.
e) Repeat step c-d for all other valves.
f) The bent needle will be connected to the pneumatic inlet of the PDMS LOC device.

**Supplemental Table 1.** Cost breakdown for polydimethylsiloxane lab-on-chip device fabrication

| Part name | Supplier | Catalog No | Quantity | Total Price | Comment |
|---|---|---|---|---|---|
| Mylar transparency mask | Artnet Pro | M13003 | 1 | $100.00 | The photomasks are used once to transfer the design to the SU-8 master wafers, and they can be reused for multiple fabrication runs. Each mask comprises a design for 3 devices. |
| Chrome mask | HTA photomask | HQ24243-1 | 1 | $700.00 | |
| Silicon wafer | University Wafer, Inc | 783 | 3 | $30.00 | - |
| Photolithography supplies (SU-8, PDMS) | Kayaku Advanced Materials Inc, Ellsworth Adhesives | NC9463827,184 SIL ELAST KIT 0.5KG | 1 unit | $33.00 | One fabricated SU-8 master wafer can be reused multiple times for fabricating new PDMS devices. |
| **Total** | | | | $863.00 | |

**Supplemental Table 2.** Cost breakdown for local IoT device

| **Part name** | **Supplier** | **Catalog No** | **Quantity** | **Total Price** | **Comment** |
|---|---|---|---|---|---|
| Raspberry Pi | Sparkfun | DEV-15446 | 1 | $45.00 | The electronics box can be reused once it is assembled. |
| 3 port Solenoid valve | SMC Pneumatics | S070M-6DC-32 | 16 | $675.00 | |
| Relay Switch | Elexol | ULN2803 Switch Board | 2 | $48.00 | |
| **Total** | | | | $768.00 | |
